# The stability of P2-layered sodium transition metal oxides in ambient atmospheres

**DOI:** 10.1038/s41467-020-17290-6

**Published:** 2020-07-15

**Authors:** Wenhua Zuo, Jimin Qiu, Xiangsi Liu, Fucheng Ren, Haodong Liu, Huajin He, Chong Luo, Jialin Li, Gregorio F. Ortiz, Huanan Duan, Jinping Liu, Ming-Sheng Wang, Yangxing Li, Riqiang Fu, Yong Yang

**Affiliations:** 1 State Key Laboratory for Physical Chemistry of Solid Surfaces, and Department of Chemistry, College of Chemistry and Chemical Engineering, Xiamen University, 361005 Xiamen, People’s Republic of China; 2 School of Energy Research, Xiamen University, 361005 Xiamen, People’s Republic of China; 3 Department of NanoEngineering, University of California San Diego, La Jolla, CA 92093 USA; 4 Department of Materials Science and Engineering, College of Materials, Xiamen University, Xiamen, 361005 Fujian, People’s Republic of China; 5 Departamento de Química Inorgánica e Ingeniería Química, Instituto Universitario de Investigación en Química Fina y Nanoquímica, Universidad de Córdoba, Campus de Rabanales, Edificio Marie Curie, E-14071 Córdoba, Spain; 6 State Key Laboratory of Metal Matrix Composites, School of Materials Science and Engineering, Shanghai Jiao Tong University, 200240 Shanghai, P. R. China; 7 School of Chemistry, Chemical Engineering and Life Science and State Key Laboratory of Advanced Technology for Materials Synthesis and Processing, Wuhan University of Technology, 430070 Wuhan, Hubei People’s Republic of China; 8 4135 Belle Meade Circle, 28012 Belmont, North Carolina USA; 9 National High Magnetic Field Laboratory, 1800 E. Paul Dirac Drive, Tallahassee, FL 32310 USA

**Keywords:** Batteries, Batteries, Batteries, Characterization and analytical techniques

## Abstract

Air-stability is one of the most important considerations for the practical application of electrode materials in energy-harvesting/storage devices, ranging from solar cells to rechargeable batteries. The promising P2-layered sodium transition metal oxides (P2-Na_x_TmO_2_) often suffer from structural/chemical transformations when contacted with moist air. However, these elaborate transitions and the evaluation rules towards air-stable P2-Na_x_TmO_2_ have not yet been clearly elucidated. Herein, taking P2-Na_0.67_MnO_2_ and P2-Na_0.67_Ni_0.33_Mn_0.67_O_2_ as key examples, we unveil the comprehensive structural/chemical degradation mechanisms of P2-Na_x_TmO_2_ in different ambient atmospheres by using various microscopic/spectroscopic characterizations and first-principle calculations. The extent of bulk structural/chemical transformation of P2-Na_x_TmO_2_ is determined by the amount of extracted Na^+^, which is mainly compensated by Na^+^/H^+^ exchange. By expanding our study to a series of Mn-based oxides, we reveal that the air-stability of P2-Na_x_TmO_2_ is highly related to their oxidation features in the first charge process and further propose a practical evaluating rule associated with redox couples for air-stable Na_x_TmO_2_ cathodes.

## Abstract (web summary)

Air-stability is a critical challenge faced by layered sodium transition metal oxide cathodes. Here, the authors depict a general and in-depth model of the structural/chemical evolution of P2-type layered oxides in air and propose an evaluation rule for the air-stability of layered sodium cathodes.

## Introduction

Practical application is always the ultimate goal for state-of-the-art technologies and devices, such as perovskite solar cells, thin-film transistors, and electrochemical batteries^1^. Air-stability is undeniably one of the key issues that researchers should consider, because any air-instable compounds must be prepared, stored and assembled in dry or even inert atmospheres, leading to an increase in expense and can even jeopardize whether they are successfully commercialized.

Sodium ion batteries (SIBs) promise the potential for large-scale grid storage, due to the high abundance and wide distribution of Na sources as compared to their lithium counterparts^2–4^. Among various sodium storage cathodes, layered sodium transition metal oxides (Na_x_TmO_2_) have gained significant attention owing to their great variety of compositions, ease of scalable preparation and high reversible specific capacity^5^. In the past forty years^6,7^, the Na_x_TmO_2_ family has been greatly enriched not only thanks to the tremendous efforts toward superior cathodes with higher energy density and more stable structures^8–10^, but has also benefited from the development of electrolytes and characterization techniques^11–14^. Nonetheless, Na_x_TmO_2_ electrodes are still haunted by three major challenges, i.e. irreversible phase transitions during cycling, insufficient electrochemical performances and air/moisture instability^5,15^. The undesired structural transformations in charge/discharge processes, such as P2-O2 in Na_0.67_Ni_0.33_Mn_0.67_O_2_^16–20^, P2-P2’ in Na_0.67_MnO_2_^10,21–23^ and O3-P3 in NaNi_0.5_Mn_0.5_O_2_^24^, can be suppressed or delayed by element substitution, thus resulting in improved cycling stability and rate capability. The contact between air-instable Na_x_TmO_2_ and moisture-air usually produces cracks^25^, electrical insulation species^26^, and hydration phases^27^, which result in a shorter lifetime and poorer rate capability of the exposed layered oxides^28–30^. Therefore, air-stability is considered an important factor to evaluate a qualified Na_x_TmO_2_ electrode^31–33^.

Recently, Manthiram’s group reported that O3-NaNi_0.7_Mn_0.15_Co_0.15_O_2_ reacts with H_2_O and CO_2_, generating NaOH, Na_2_CO_3_, Na_2_CO_3_∙H_2_O and NiO on the particle surfaces and leading to declined electrochemical performances^26^. However, these degradation (Ni loss) reactions have not yet been extensively observed in Ni-poor/Ni-free Na_x_TmO_2_ materials, whether exposed to air or immersed in water^34,35^. Unlike degradation, H_2_O insertion is widely encountered in P2-Na_x_TmO_2_ electrodes and can be easily distinguished by X-ray diffraction (XRD), because the intercalation of H_2_O expands the interlayer distance of the Na^+^ layers from ~5 to ~7 Å and even up to ~9 Å^36–38^. Kubota et al. proposed the Na^+^/H^+^ exchange mechanisms in O3-type NaMeO_2_ oxides^39^ and Rojo’s group further identified the presence of H^+^ in the Na^+^ layers using a neutron powder diffraction technique^30^. In addition, in the presence of H_2_O, CO_2_ was believed to get inserted into the transition metal layers of P2-Na_2/3_Fe_0.5_Mn_0.5_O_2_^28^. These works provide valuable insights into the structural evolutions of Na_x_TmO_2_ upon air-exposure. However, rational connections between these intertwined reactions, especially as to why and when the water molecules insert into the Na^+^ layers, and which factor determines the extent of the structural transformation have not been thoroughly studied yet.

Fundamental understanding of structural/chemical evolutions in moisture is not only critical for the employment of Na_x_TmO_2_ as electrodes in both organic and aqueous SIBs, but also provides guidance to the fabrication and application of layered transition metal oxides in other battery systems, such as the favorable Ni-rich^40–43^ cathodes for lithium ion batteries. For layered sodium-based oxides, it is widely recognized that the air-instability in P2-type oxides is much more severe than that in O3-type Na_x_MnO_2_^5^. Herein, based on the P2-Na_0.67_MnO_2_ and P2-Na_0.67_Ni_0.33_Mn_0.67_O_2_ oxides, we unearth the underlying science that triggers the hydration of P2-Na_x_TmO_2_, apply unique solid-state nuclear magnetic resonance techniques (ss-NMR) to provide solid evidence for H^+^ insertion, analyze critical factors that influence the hydration, and outline the structural and chemical evolution mechanisms of P2-Na_x_TmO_2_ oxides upon air exposure. Importantly, the surface-sensitive time-of-flight secondary ion mass spectroscopy (TOF-SIMS) results reveal that the CO_2_ cannot insert into the layered structure. Our results indicate that Na^+^/H^+^ exchange, rather than O_2_ oxidation, dominates the compensation of extracted Na^+^ and the hydration is closely related to the contents of remaining Na^+^ ions in the structure. The critical sodium content ***n***_***c***_ is therefore proposed to evaluate whether H_2_O is able to intercalate into the sodium layers. In addition, the study of a series of P2-Na_x_TmO_2_ oxides indicates that the air-stability is closely associated with the voltage features of the charge process in the first cycle, and a practical principle related to the redox couple in the 1^st^ cycle is thus proposed to evaluate the air-stability of Na_x_TmO_2_. These new insights into the degradation mechanisms upon air-exposure will facilitate the development of practical layered Li/Na/K transition metal oxides.

## Results

### Features of hydration phases

**Fig. 1:**
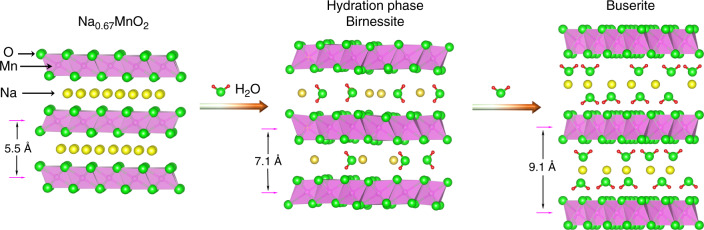
Structural illustration of water insertion. Schematic illustration of P2-Na_0.67_MnO_2_, birnessite, and buserite phases.

To investigate the air-stable mechanisms, the structures and characterizations of hydration phases should be clarified first. The Na_0.67_MnO_2_ sample attained by solid-state reaction exhibits a typical P2 structure (space group: *P6*_*3*_*/mmc*) with a layer spacing of ~5.5 Å (Fig. 1), according to the powder X-ray diffraction (XRD) patterns in Supplementary Fig. 1a. The commonly reported hydration impurity phase in the Na_x_TmO_2_ is birnessite^27,30,37^, whose structure is very similar to that of P2-Na_0.67_MnO_2_, except the presence of extra water molecules in the sodium layers and a broader interlayer distance of ~7.1 Å. With further H_2_O insertion, the buserite phase with layer spacing of ~9.1 Å can be identified (Fig. 1 and Supplementary Fig. 1a). In addition to the difference in the interlayer spacing, the birnessite and buserite phases possess a different pattern of Na^+^ and H_2_O arrangements in the sodium layers. For birnessite ({Na_0.30_•(H_2_O)_0.45_}MnO_2_), according to the Rietveld refinement results^44^ (Supplementary Table 1 and Fig. 1b) and the neutron powder diffraction patterns (NPD)^45^, the O ion of inserted H_2_O molecule locates at the same site of Na^+^; while for the highly hydrated buserite ({Na_0.24_•(H_2_O)_2.1_}MnO_2_), the Na^+^ ions are sandwiched by the inserted H_2_O molecules^46,47^, as illustrated schematically in Fig. 1. Since buserite is seldom encountered in the moisture-exposed Na_x_TmO_2_, the hydration phase discussed below is referred to birnessite phase unless otherwise specified. It should be further pointed out that the Na^+^ content in hydrated Na_0.67_TmO_2_ sample is <0.67 due to the Na^+^ loss upon air-exposure, as shown in the following sections. XRD, NPD and ^23^Na magic-angle-spinning nuclear magnetic resonance spectroscopy (MAS NMR, Supplementary Figs. 1–3 and Note 1) are three powerful techniques to identify the hydration impurities (birnessite) in Na_x_TmO_2_ oxides, by characterizing the structural transitions, i.e. increased interlayer spacings. Fourier-transform infrared spectroscopy (FTIR) and ^23^Na{^1^H} rotational-echo double-resonance (REDOR)^48^ can be used to detect the existence of water molecules and protons in the Na_x_TmO_2_ compounds, respectively (Supplementary Fig. 4 and Notes 1, and 2).

### The structural transitions of the moisture-exposed P2-Na_0.67_TmO_2_

**Fig. 2:**
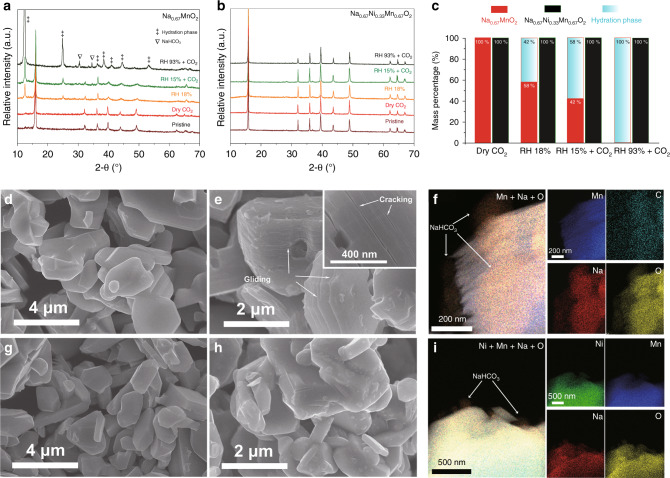
The structural transitions of P2-Na_0.67_TmO_2_ upon air-exposure. The XRD patterns of **a** Na_0.67_MnO_2_ and **b** Na_0.67_Ni_0.33_Mn_0.67_O_2_ samples exposed in different atmosphere. **c** The refinement results of exposed Na_0.67_MnO_2_ and Na_0.67_Ni_0.33_Mn_0.67_O_2_ samples with a two-phase model. The SEM images of **d** pristine and **e** RH 93% + CO_2_ exposed Na_0.67_MnO_2_ powder, **f** The EDS mapping results of exposed Na_0.67_MnO_2_, indicating that NaHCO_3_ is formed on the particles’ surface. The SEM images of **g** pristine and **h** RH 93% + CO_2_ exposed Na_0.67_Ni_0.33_Mn_0.67_O_2_ powder. **i** The EDS mapping results of RH 93% + CO_2_ exposed Na_0.67_Ni_0.33_Mn_0.67_O_2_ samples, NaHCO_3_ particles are also observed on the surface of exposed Na_0.67_Ni_0.33_Mn_0.67_O_2_. The exposure time of the above samples are 3 days.

To investigate the structural changes of P2-Na_x_TmO_2_ during the air-exposure, Na_0.67_MnO_2_ and Na_0.67_Ni_0.33_Mn_0.67_O_2_ were selected as model compounds for air-instable and air-stable electrodes, respectively^27^, to be exposed in different atmospheres of dry CO_2_, relative humidity (RH) 18% (without CO_2_), RH 15% + CO_2_ (with presence of CO_2_), and RH 93% + CO_2_ for 3 days (“Methods” part). As shown in the XRD patterns (Fig. 2a, b) and inductively coupled plasma-atomic emission spectrometry (ICP-AES) results (Supplementary Table 2), the targeted materials were successfully prepared. It can be observed that all XRD peaks of Na_0.67_MnO_2_ remain unchanged when placed in the dry CO_2_ atmosphere (Fig. 2a), revealing that water is an inevitable component to destabilize Na_0.67_TmO_2_. At the atmospheres of RH 18% and RH 15% + CO_2_, the hydration peaks (labeled with ‡) emerge and their intensities increase with the presence of CO_2_. With further increase in the relative humidity (RH 93% + CO_2_), all of the XRD peaks can be indexed to the hydration phase and NaHCO_3_ (labeled with ∇), indicating that the Na_0.67_MnO_2_ is totally hydrated and partial Na^+^ diffuses out from the sodium layers. For Na_0.67_Ni_0.33_Mn_0.67_O_2_, no obvious hydration peaks can be identified in all of the exposed samples (Fig. 2b), suggesting Na_0.67_Ni_0.33_Mn_0.67_O_2_ is more stable than Na_0.67_MnO_2_ in moist atmospheres. To quantify the hydration extent of the exposed samples, Rietveld refinements on the XRD patterns were conducted with a two-phase model and the results are shown in Fig. 2c. The mass ratios of hydrated phases in the exposed Na_0.67_MnO_2_ samples are 0%, 42%, 58%, and 100% at the atmosphere of dry CO_2_, RH 18%, RH 15% + CO_2_, and RH 93% + CO_2_, respectively, revealing that P2-Na_x_TmO_2_ components are more vulnerable to H_2_O with both the existence of CO_2_ and the increment of relative humidity.

Figure 2d–i depicts the morphological change of Na_0.67_MnO_2_ and Na_0.67_Ni_0.33_Mn_0.67_O_2_ powder after the exposure to the atmosphere of RH 93% +  CO_2_ for 3 days. Scanning electron microscopy (SEM) images in Fig. 2d, g show that the surface of as-prepared Na_0.67_MnO_2_ and Na_0.67_Ni_0.33_Mn_0.67_O_2_ particles are clean and smooth. After exposure, severe delamination damages and a massive of intragranular cracks can be observed in hydrated Na_0.67_MnO_2_ sample (Fig. 2e), as a result of the significant volume expansion of ~30% during the hydration process. Besides structural changes, NaHCO_3_ particles are found on the surface of hydrated Na_0.67_MnO_2_, as shown in the energy dispersive X-ray spectroscopy (EDS) elemental mapping results (Fig. 2f). Moreover, the crystals with very regular morphologies can be observed in the exposed-Na_0.67_MnO_2_ powder (Supplementary Fig. 5a, b), which should be unambiguously identified to NaHCO_3_, according to the FTIR (Supplementary Fig. 4a) and EDS mapping results in Supplementary Fig. 5c. The formation of sodium salt is also observed in the air-exposed electrodes. As shown in Supplementary Fig. 6, sodium bicarbonate particles with nanoflakes morphology appear at the surface of the active materials. For exposed-Na_0.67_Ni_0.33_Mn_0.67_O_2_ sample, as shown in Fig. 2h, a rough surface is formed and there is no evidence of crack and layer glide, indicating that the morphological change of Na_0.67_Ni_0.33_Mn_0.67_O_2_ during exposure is more moderate than the Na_0.67_MnO_2_ sample (Fig. 2e). However, the EDS mapping results in Fig. 2i reveal that the NaHCO_3_ particles are formed on the surface of exposed Na_0.67_Ni_0.33_Mn_0.67_O_2_, indicating the Na^+^ loss in Na_0.67_Ni_0.33_Mn_0.67_O_2_ compound during exposure. In summary, the above XRD, SEM and EDS results indicate that Na_0.67_MnO_2_ samples experience chemical transitions upon air-exposure, and Na_0.67_Ni_0.33_Mn_0.67_O_2_ changes as well.

Several influential factors especially relative humidity must be taken into consideration during the air-exposure experiments, soaking the samples in water is therefore a widely accepted testing procedure for confirming the stability of layered Na_x_TmO_2_ oxides^31^. Moreover, water-stability is an important metric of Na_x_TmO_2_ when applied as aqueous battery electrodes. Therefore, the structural evolutions of Na_0.67_MnO_2_ and Na_0.67_Ni_0.33_Mn_0.67_O_2_ samples in water have also been investigated. The results in Supplementary Figs. 7 and 8 and detailed analysis in Supplementary Note 3 suggest that the structural transformation mechanisms of P2-Na_x_TmO_2_ in water are similar to that in moist air.

### The role of CO_2_

**Fig. 3:**
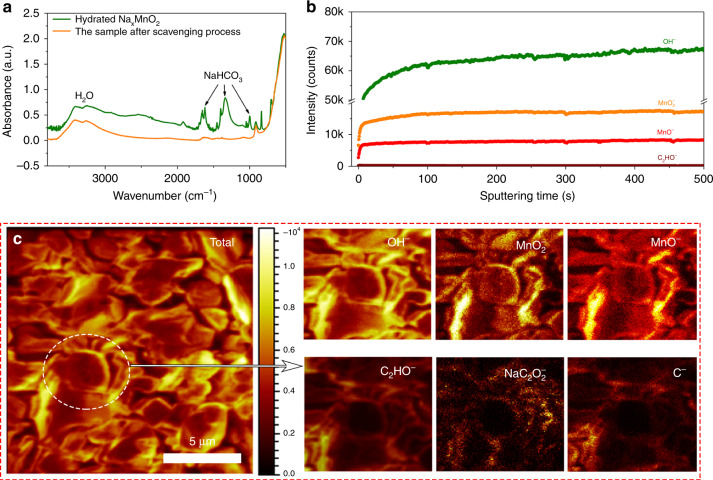
TOF-SIMS results of the hydrated Na_0.67_MnO_2_ sample. **a** the comparison of FTIR spectra of the hydrated Na_0.67_MnO_2_ and hydrated Na_0.67_MnO_2_ after the scavenging process, indicating that after scavenging, most of the sodium (bi)carbonate impurities was removed. **b** TOP-SIMS spectra of OH^−^, MnO^−^, MnO_2_^−^, and C_2_HO^−^ secondary ion fragments over 500 s Cs^+^ sputtering along the depth profile of the sample after scavenging processes. **c** TOF-SIMS chemical mapping of the hydrated Na_0.67_MnO_2_ after the scavenging process, showing the distribution of OH^−^, MnO_2_^−^, MnO^−^, C_2_HO^−^, NaC_2_O_2_^−^, and C^−^ secondary ions.

The presence of CO_2_ makes a big difference in the structural transformations of P2-Na_x_TmO_2_ upon moisture-exposure (Fig. 2c). To characterize the functionality of CO_2_, we applied the TOF-SIMS to ascertain whether CO_2_ intercalates into the bulk of the P2-Na_x_TmO_2_ during hydration^5,26–28,30^. In order to eliminate the influence of the surficial sodium bicarbonate species (Fig. 2f and Supplementary Fig. 4), we adopted a scavenging process (see details in the Methods section). The FTIR spectra in Fig. 3a reveal that NaHCO_3_ in the hydrated Na_0.67_MnO_2_ sample is successfully removed. TOF-SIMS depth profiles acquired on the scavenged powder show that the H_2_O (OH^−^) and Mn ions (MnO^−^ and MnO_2_^−^) appear at both surface and bulk of the powder (Fig. 3b), in good agreement with our refinement results (Supplementary Table 1). The signal intensity of C_2_HO^−^ in the bulk (~165 a.u., Supplementary Fig. 9 and Note 4) is only 2.5 ‰ compared to that of the prevailed OH^−^ (65k a.u.). Considering that the water content in hydrated Na_0.67_MnO_2_ is ~0.45 mol per chemical formula unit (Supplementary Table 1), it is therefore reasonable to conclude that the C atoms in the bulk of the hydrated samples are negligible. Furthermore, the cross-sectional TOF-SIMS chemical mapping images of the tested secondary particles (Fig. 3c) show that the intensity of C-related species (e.g. C_2_HO^−^, NaC_2_O_2_^−^, C^−^) is much lower than OH^−^ and Mn-based ions and the remaining carbonate trace is mainly distributed on the surface rather than the bulk of the particles. This fact coincides well with the depth profile results in Fig. 3b and Supplementary Fig. 9. Therefore, the function of CO_2_ is to increase the acidity at the particle’s surface and accelerate the Na^+^ loss rather than insert into the bulk of the Na_x_TmO_2_ phases.

### The critical sodium contents (*n*_*c*_) for hydration

**Fig. 4:**
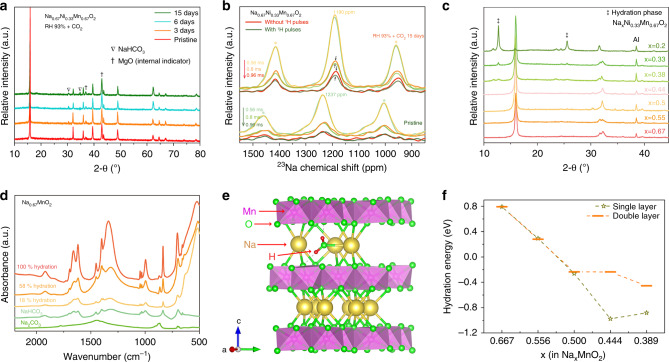
The structural and chemical evolution mechanisms upon air-exposure. **a** The XRD evolutions of Na_0.67_Ni_0.33_Mn_0.67_O_2_ powder exposed at RH 93% + CO_2_ atmosphere for different times (MgO was used as internal indicator). **b** ^23^Na{^1^H} REDOR-dephased ^23^Na MAS NMR spectra (MAS rate: 25 kHz) and **c** The XRD patterns of exposed Na_x_Ni_0.33_Mn_0.67_O_2_ electrodes with various Na^+^ content x. **d** The FTIR spectra of Na_0.67_MnO_2_ samples with various hydration degrees. **e** The structure model of single layer Na^+^ loss structure for calculating hydration energies. **f** The calculated hydration energies for Na_x_MnO_2_ at various sodium contents x.

Although Na_0.67_Ni_0.33_Mn_0.67_O_2_ has been reported to be one of the most air-stable P2-Na_x_TmO_2_ oxides, the chemical change is observed in the EDS mapping as demonstrated in Fig. 2i. Furthermore, as shown in Fig. 4a, with longer exposure time, the intensity of the NaHCO_3_ diffraction peaks increases while the (002) diffraction peak shifts to lower 2-theta (Supplementary Fig. 10), suggesting the gradual extraction of Na^+^ from Na_0.67_Ni_0.33_Mn_0.67_O_2_ during exposure. To validate the identification of NaHCO_3_, the pristine and exposed Na_0.67_Ni_0.33_Mn_0.67_O_2_ samples are further scrutinized by FTIR. As shown in Supplementary Fig. 11, the characteristic peaks located at 600-2000 cm^−1^ in the FTIR spectrum of the exposed Na_0.67_Ni_0.33_Mn_0.67_O_2_ in RH 93% + CO_2_ fit well with that of NaHCO_3_. The absence of the O–H stretching signal between ~2500 and 3500 cm^−1^ suggests that no H_2_O intercalates into the sodium layers of Na_0.67_Ni_0.33_Mn_0.67_O_2_, which is highly consistent with the XRD results. In addition, as shown in Fig. 4b and Supplementary Fig. 12, the ^23^Na MAS NMR signal of Na_0.67_Ni_0.33_Mn_0.67_O_2_ shifts to the upper field by 47 ppm, corresponding to the expansion of c parameter (Supplementary Fig. 10). It is worth noting that the intensity of ^23^Na{^1^H} REDOR signal for the exposed Na_0.67_Ni_0.33_Mn_0.67_O_2_ sample decreases with ^1^H irradiation as a function of the spin-echo time, as compared to that without ^1^H irradiation. This result confirms that H^+^ ions are in close proximity to the sodium atoms in the structure of the exposed Na_0.67_Ni_0.33_Mn_0.67_O_2_ sample. Thus, it is more likely that the H ions replace those lost Na^+^, indicative of the Na^+^/H^+^ exchange mechanism. Moreover, given that the exposed electrodes usually exhibit improved open circuit potential^26^, another charge compensation mechanism, i.e. the oxidation of transition metal ions by O_2_ has been also proposed^5^. To validate which mechanism dominates the Na^+^ loss process, X-ray absorption spectroscopy (XAS) was carried out. According to the previous results^8,20,49^, during the first charge process of Na_0.67_Ni_0.33_Mn_0.67_O_2_ electrode within 2.0–4.4 V (vs. Na^+^/Na), Mn^4+^ ions remain stable and the oxidation/reduction of Ni ions compensates the electrochemical extraction/insertion of Na^+^. As shown in Supplementary Fig. 13a, both position and shape of the Ni K-edge remain unchanged, indicating that the valence state of Ni ions in the exposed Na_0.67_Ni_0.33_Mn_0.67_O_2_ is nearly the same as in the pristine sample. The valence state of Mn ions in pristine and hydrated Na_0.67_MnO_2_ samples has been also investigated by XAS. The pre-edge peak and main edge of Mn K-edge XAS correspond to 1*s* → 3*d* and 1*s* → 4*p* transitions, respectively. However, the structural changes during hydration (Supplementary Fig. 13b) severely complicate the chemical shift of the main edge such that it becomes difficult to determine the valence state of Mn ions. Thus the pre-edge region could provide more reliable valent-state information^50^. As can be seen from Supplementary Fig. 13b, the splitting and intensity of pre-edge peaks of Mn ions are similar for all of the pristine, partially hydrated and totally hydrated samples, indicating that the changes of the valence state of Mn ions are negligible during hydration. The XAS results suggest that Na^+^/H^+^ exchange might be the main reaction that compensates for the extracted Na^+^ in the moisture-exposed Na_x_TmO_2_. To confirm this conclusion, we stored the Na_0.67_MnO_2_ sample in three different atmospheres for 3 days, e.g. O_2_, RH 93% + CO_2_ with the presence of O_2_, and RH 93% + CO_2_ without O_2_. The XRD patterns in Fig. 2a and Supplementary Fig. 13c suggest that O_2_ makes smaller difference than both CO_2_ and water to the structural changes of Na_x_TmO_2_.

To gain deeper insight into the structural/chemical transformation mechanisms of P2-Na_x_TmO_2_ materials upon air-exposure, a series of Na_x_Ni_0.33_Mn_0.67_O_2_ electrodes with different Na^+^ contents were prepared by extracting Na^+^ electrochemically, and then exposed at RH 93% + CO_2_ atmosphere for 3 days. As shown in Fig. 4c, the hydration phase is absent at Na_x_Ni_0.33_Mn_2/3_O_2_ electrodes within sodium contents of 0.67 ≤ *x* ≤ 0.44. In *x* = 0.38, the diffraction peaks of the hydration phase appear and its intensity increases with the further decrease of Na^+^ contents. This result implies that hydration highly depend on the Na^+^ contents. Specifically, if the sodium content ***x*** is lower than a critical sodium content ***n***_***c***_ (0.38–0.44 for Na_x_Ni_0.33_Mn_2/3_O_2_ electrode), P2-Na_x_TmO_2_ materials are vulnerable for hydration and vice versa. The correlation between hydration degree and sodium contents ***x*** is also observed in Na_x_MnO_2_ compounds. As representatively shown in Fig. 4d, the intensity of NaHCO_3_ absorption peaks are positively associated with the hydration degrees of Na_0.67_MnO_2_ sample.

The density function theory (DFT) calculation was carried out to further understand the critical sodium content ***n***_***c***_. As shown in Fig. 4e and Supplementary Fig. 13d, two different sodium extraction models of single layer and double layer were considered, which correspond to the staging and random sodium extraction mechanisms, respectively^15^. The energy difference between P2-Na_x_TmO_2_ with and without water molecules is defined as hydration energy. DFT calculation results in Fig. 4f show that when *x* > 0.52, the hydration energy of Na_x_MnO_2_ is higher than 0 eV, suggesting that water molecules cannot get inserted into the material. When the sodium content is lower than 0.52, the hydration energy is lower than 0 eV, thus the hydrated structure is more stable than the un-hydrated structure. The foregoing energy analysis provides a chemical basis for understanding the sodium-content-dependent property in the hydration of Na_x_MnO_2_ samples. Due to the low formation energy, the layered Na_0.67_TmO_2_ oxides will lose Na^+^ spontaneously once in contact with moist air. When the sodium contents are lower than ***n***_***c***_, there is a strong repulsion between adjacent oxide layers (TmO_2_) of P2-Na_x_TmO_2_ oxides, and the insertion of H_2_O could lower the energy of Na_0.67-x-z_H_x_TmO_2_ (x and z correspond to the Na^+^ loss that compensated by Na^+^/H^+^ exchange and O_2_ oxidation, respectively) due to the shielding effect of the water molecule and the expansion of sodium layer spacings. The above results also proved and interpreted the fact that hydration takes place after the extraction of Na^+^ during air-exposure.

Based on these results and analyses, the structural and chemical transformations of exposed P2-Na_0.67_TmO_2_ to different ambient atmospheres are summarized in Eqs. (1–4).

In the atmosphere without CO_2_:

1 $$x \cdot {\rm{H}}_2{\rm{O}} + {\rm{Na}}_{0.67}{\rm{TmO}}_2 = {\rm{Na}}_{0.67 - x}{\rm{H}}_x{\rm{TmO}}_2 + x \cdot {\rm{NaOH}}$$

In the atmosphere with scarce CO_2_:

2 $$	x \cdot {\rm{CO}}_2 + x \cdot {\rm{H}}_2{\rm{O}} + 2 \cdot {\rm{Na}}_{0.67}{\rm{TmO}}_2 = \, 2 \cdot {\rm{Na}}_{0.67 - x}{\rm{H}}_x{\rm{TmO}}_2 \\ 	+ x \cdot {\rm{Na}}_2{\rm{CO}}_3$$

In the atmosphere with abundant CO_2_:

3 $$	x \cdot {\rm{CO}}_2 + x \cdot {\rm{H}}_2{\rm{O}} + {\rm{Na}}_{0.67}{\rm{TmO}}_2 =\, {\rm{Na}}_{0.67 - x}{\rm{H}}_x{\rm{TmO}}_2 \\ 	+ x \cdot {\rm{NaHCO}}_3$$

If the sodium content in the electrode is lower than ***n***_***c***_:

4 $${\rm{Na}}_{0.67 - x}{\rm{H}}_x{\rm{TmO}}_2 + y \cdot {\rm{H}}_2{\rm{O}} = \left[ {{\rm{Na}}_{0.67 - x}{\rm{H}}_x({\rm{H}}_2{\rm{O}})_y} \right]{\rm{TmO}}_2$$

It should be emphasized that although Tm oxidation is not included in the proposed charge-compensation mechanisms due to lacking of direct spectroscopic evidences, there is still a high possibility that a minority of charge is compensated by the valence change of Tm ions. In the near future, more techniques, especially the state-of-the-art s-XAS characterizations are expected to obtain a deeper understanding of the charge-compensation mechanisms of Na^+^ extraction in moisture-exposed Na_x_TmO_2_.

### The influence of structural changes on the electrochemical performances of P2-Na_x_TmO_2_

**Fig. 5:**
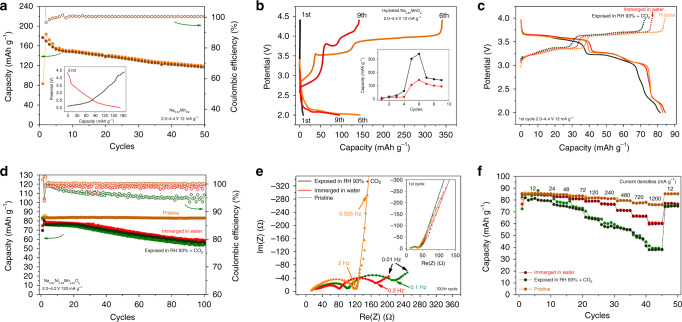
Electrochemical performances of air-exposed Na_0.67_MnO_2_ and Na_0.67_Ni_0.33_Mn_0.67_O_2_. **a** The cycling performance of pristine Na_0.67_MnO_2_, the inset shows the charge-discharge curve at the 2nd cycle. **b** The charge-discharge curves and cycling stability of totally hydrated Na_0.67_MnO_2_ sample. **c**–**f** The electrochemical performances within the voltage range of 2.0–4.0 V of three Na_0.67_Ni_0.33_Mn_0.67_O_2_ samples (pristine Na_0.67_Ni_0.33_Mn_0.67_O_2_, Na_0.67_Ni_0.33_Mn_0.67_O_2_ samples immersed in water for 20 days and exposed in RH 93% + CO_2_ for 3 days). **c** The initial charge-discharge curves at 12 mA g^−1^. **d** The cycling stability of the three samples at 120 mA g^−1^ (after two formation cycles at 12 mA g^−1^). **e** The comparison of the impedance responses at the 1st and 100th cycles with frequency varied from 100 kHz to 10 mHz. **f** The rate capability of three samples at the voltage range of 2.0–4.0 V.

In this section, we carefully investigate the influences of the above structural and chemical changes on the electrochemical performances of Na_0.67_MnO_2_ and Na_0.67_Ni_0.33_Mn_2/3_O_2_ electrodes. As shown in Fig. 5a, the pristine Na_0.67_MnO_2_ shows a high initial discharge capacity of 176 mAh g^−1^ at the current density of 12 mA g^−1^ within 2.0–4.4 V. After 50 cycles, 66 % of the initial capacity was retained, which coincides well with the previous results^10,23^. For the hydrated Na_0.67_MnO_2_ electrode, the reversible capacity is negligible at the initial cycles. In the 9^th^ cycle, although the discharge capacity increased to ~150 mAh g^−1^, abnormal electrochemistry, such as long charging plateaus, large voltage hysteresis and low coulombic efficiency are also observed. These deviant electrochemical performances indicate that the large amount of H_2_O in the hydrated electrode has a pernicious influence on the organic-electrolyte based batteries.

The electrochemical performances of pristine Na_0.67_Ni_0.33_Mn_0.67_O_2_, Na_0.67_Ni_0.33_Mn_0.67_O_2_ exposed in RH 93% + CO_2_ for 3 days (denoted as exposed-Na_0.67_Ni_0.33_Mn_0.67_O_2_), and Na_0.67_Ni_0.33_Mn_0.67_O_2_ immersed in water for 20 days (denoted as immersed-Na_0.67_Ni_0.33_Mn_0.67_O_2_) are presented in Fig. 5c–f. As shown in Fig. 5c, the pristine Na_0.67_Ni_0.33_Mn_0.67_O_2_, exposed-Na_0.67_Ni_0.33_Mn_0.67_O_2_, and immersed-Na_0.67_Ni_0.33_Mn_0.67_O_2_ samples have very similar initial charge-discharge curves, suggesting that their structure and redox center are nearly identical^8^. However, the initial charge capacities of the exposed-Na_0.67_Ni_0.33_Mn_0.67_O_2_ (70 mAh g^−1^) and immersed-Na_0.67_Ni_0.33_Mn_0.67_O_2_ (77 mAh g^−1^) are lower than that of the pristine Na_0.67_Ni_0.33_Mn_0.67_O_2_ (85 mAh g^−1^), further confirming the loss of Na^+^ ions during the exposure and immersing processes. Fig. 5d compares the cycling stability of the three electrodes. It can be observed that the exposed-Na_0.67_Ni_0.33_Mn_0.67_O_2_ exhibits much lower coulombic efficiency than the pristine Na_0.67_Ni_0.33_Mn_0.67_O_2_, due to the influence of the sodium bicarbonate on the surface of exposed-Na_0.67_Ni_0.33_Mn_0.67_O_2_ particles. At the voltage range of 2.0–4.0 V, the pristine Na_0.67_Ni_0.33_Mn_0.67_O_2_ material shows no evidence of capacity degradation after 100 cycles. In contrast, the immersed-Na_0.67_Ni_0.33_Mn_0.67_O_2_ and exposed-Na_0.67_Ni_0.33_Mn_0.67_O_2_ samples decay fast in capacity, and retain low discharge capacity of 56 and 53 mAh g^−1^, corresponding to the capacity retention of 74% and 70%, respectively. To get more insight into the degradation mechanisms, the electrochemical impedance spectra (EIS) have been employed to study the resistance changes. The interface and charge-transfer resistances were estimated based on the equivalent circuit in Supplementary Fig. 14 and summarized in Supplementary Table 3. In the first cycle, the electrode resistance of the three samples is quite similar (the inset in Fig. 5e). After 100 cycles, the rapid increase of the surface resistance (R_SEI_) and charge transfer resistance (R_C, CT_) for the exposed-Na_0.67_Ni_0.33_Mn_0.67_O_2_ and immersed-Na_0.67_Ni_0.33_Mn_0.67_O_2_ (Fig. 5e) samples indicates that the decomposition layer on the surface and the proton ions in the structure accelerate the depletion of the electrolyte thus blocking the diffusion of Na^+^ ions. The comparison of rate capabilities shows a similar trend of cycling stability. As shown in Fig. 5f, the pristine Na_0.67_Ni_0.33_Mn_0.67_O_2_ electrode exhibits higher capacities than the immersed and exposed samples at all rates. The above electrochemical results demonstrate that exposure to moist air and immersion in water cause undesired structural changes and result in deteriorated electrochemical performances of P2-Na_x_TmO_2_ samples.

### The structural transformation of hydrated phases upon calcination in air

**Fig. 6:**
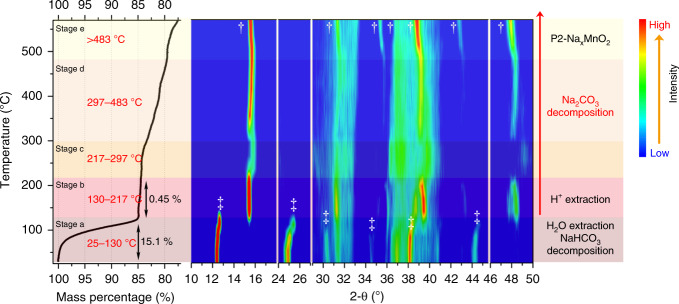
Temperature-resolved in situ XRD of the totally hydrated Na_0.67_MnO_2_. The temperature ranges from 25 to 570 °C. The TGA curves are shown at the left. Hydrated and P2 phases are labeled with “‡” and “†”, respectively.

We have concluded that the sodium extraction (mainly compensated by Na^+^/H^+^ exchange) and H_2_O insertion are two continuous structural transition processes during the exposure of P2-Na_x_TmO_2_ samples, which usually impair their intrinsic electrochemical performances. The following questions need to be clarified: (i) whether or not these exposed samples can be recovered to their original structure and how, and (ii) what is the stoichiometry of hydrated phases (birnessite)? Therefore, in this section, the structural transitions of hydrated samples at the temperature range of 25–570 °C were investigated by in situ variable-temperature XRD technique. As shown in Fig. 6, five different stages can be observed in the in situ variable-temperature XRD pattern of the hydrated Na_0.67_MnO_2_. The mass loss at stage *a* (25–130 °C) is ~15.1%, which includes decomposition of NaHCO_3_^51^ and extraction of water. At this stage, the hydration structure is well maintained. From stage *a* to stage *b*, the hydrated phase is most probably transformed into a protonated phase (Supplementary Fig. 15 and Note 5) by two-phase reaction mechanisms at ~130 °C. The crystallinity of the Na_0.67-x-z_MnO_2_ phase at stage *c* (217–297 °C) is much lower than stage *b*, which might be resulted from the loss of protons. In addition, the sodium carbonate begins to decompose and results in the mass loss in stage *c*. With further increase in temperature, the crystallinity of Na_0.67-x-z_MnO_2_ increases, as shown in stage *d*. It should be pointed out that the decomposition temperature of Na_2_CO_3_ is lower than 800 °C due to the small sizes of the Na_2_CO_3_ particles (Fig. 2 and Supplementary Fig. 5) and the catalytic effect of transition metal oxides on reducing the energy requirement of Na_2_CO_3_ decomposition^52^. When the temperature is higher than 483 °C, the (100) and (103) peaks (at ~35° and ~43°, respectively) of the P2 phase emerge gradually, indicating the P2-Na_0.67_MnO_2_ phase gradually recovers at stage *e*. However, the mass loss of 0.45% during 130–217 °C corresponds to *x* = 0.61 in Na_0.67-x-z_H_x_MnO_2_, exceeding the amount of lost Na^+^ ions (*x* + *z* ≈ 0.39, Supplementary Table 4), suggesting that the decomposition of Na_2_CO_3_ begins at stage *b* (130–217 °C). In conclusion, with the increase of temperature, the hydrated Na_0.67_MnO_2_ sample undergoes dehydration and NaHCO_3_ decomposition (70–130 °C), deprotonation (130–217 °C), Na_2_CO_3_ decomposition (>130 °C) and the recrystallization of P2 phase (>483 °C) process. In our recent work^23^, we demonstrate that Na_0.67_Zn_0.1_Mn_0.67_O_2_ is a promising Na-ion battery cathode with outstanding cycling stability and better air-stability than Na_0.67_MnO_2_. Therefore, we also performed the in situ variable-temperature XRD of totally hydrated Na_0.67_Zn_0.1_Mn_0.9_O_2_ powder. Obviously, the structural transformation of the hydrated Na_0.67_Zn_0.1_Mn_0.9_O_2_ in Supplementary Fig. 16 is different from that of the hydrated Na_0.67_MnO_2_. From the hydration phase (stage *a*) to the protonated phase (stage *b*), the combined mechanism of two-phase and solid-solution reactions can be recognized. Moreover, the temperature range of deprotonation of the hydrated Na_0.67_Zn_0.1_Mn_0.9_O_2_ sample is elusive and the temperature of the recrystallization of P2-type Na_0.67_Zn_0.1_Mn_0.9_O_2_ phase (376 °C) is lower than that of Na_0.67_MnO_2_ (483 °C). The above results indicate that if the Na_0.67_TmO_2_ samples undergo sodium-loss and further hydration processes upon air-exposure, high-temperature annealing can be used to recover their original structures (the results of verification test are shown in Supplementary Fig. 17 and Note 6). Although the detailed structural transformation mechanisms during annealing are highly dependent on the stoichiometry of the exposed Na_0.67_TmO_2_, the general structural/chemical evolution processes are concluded in Eqs. (5–7) (Supplementary Note 7 and 8).

30–130 °C:

5 $$2x \cdot {\rm{NaHCO}}_3 = x \cdot {\rm{Na}}_2{\rm{CO}}_3 + x \cdot {\rm{H}}_2{\rm{O}} + x \cdot {\rm{CO}}_2$$

6 $$[{\rm{Na}}_{0.67 - x}{\rm{H}}_x({\rm{H}}_2{\rm{O}})_y]{\rm{TmO}}_2 = y \cdot {\rm{H}}_2{\rm{O}} + [{\rm{Na}}_{0.67 - x}{\rm{H}}_x]{\rm{TmO}}_2$$

130–900 °C:

7 $$2 \cdot \left[ {{\rm{Na}}_{0.67 - x}{\rm{H}}_x} \right]{\rm{TmO}}_2 + x \cdot {\rm{Na}}_2{\rm{CO}}_3 = \, x \cdot {\rm{H}}_2{\rm{O}} + x \cdot {\rm{CO}}_2 \\ + 2 \cdot {\rm{Na}}_{0.67}{\rm{TmO}}_2$$

According to the structural/chemical evolution processes revealed by the above in situ XRD patterns, the chemical formulas of several hydrated Na_0.67_TmO_2_ materials, including Na_0.67_MnO_2_, Na_0.67_Al_0.1_Mn_0.9_O_2_, Na_0.67_Cu_0.1_Mn_0.9_O_2_, Na_0.67_Zn_0.1_Mn_0.9_O_2_, and Na_0.67_Zn_0.2_Mn_0.8_O_2_, are identified based on the TGA analysis (see detailed information in Supplementary Figs. 18 and 19, Table 4, Supplementary Eqs. (1–8), and Supplementary Note 8). ICP-AES analyses in Supplementary Table 4 are in good agreement with the chemical formulas of hydration phases. The results suggest that the contents of inserted H_2_O vary greatly from ~0.1 to 0.45 depending on the stoichiometry of pristine materials.

### The comprehensive structural/chemical evolution mechanisms upon air-exposure

**Fig. 7:**
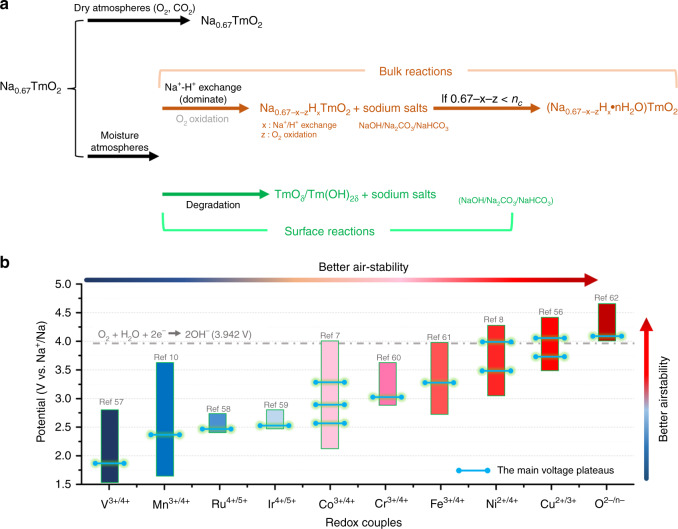
Reaction pathways and evaluation rule. **a** Proposed reaction pathways for the moisture-exposed Na_0.67_TmO_2_ materials. **b** The comparison of the main redox couples in layered sodium transition metal oxides.

Based on our analysis above, we summarize the structural and chemical transitions of the P2-Na_0.67_TmO_2_ components upon air exposure in Fig. 7a. At the initial stage, because of the low formation energy of layered Na_0.67_TmO_2_ oxides, the sodium ions are lost from the bulk and form amorphous Na_2_CO_3_/NaHCO_3_ layer on the surface of particles or even Na_2_CO_3_/NaHCO_3_ crystals with charge-compensation mechanism of Na^+^/H^+^ exchange. The formation energy increases with the extraction of Na^+^ ions and finally the P2-Na_x_TmO_2_ oxides are stable at the sodium content of 0.67-x-z in a specific environment. If the remaining sodium content 0.67-x-z is lower than the critical sodium content ***n***_***c***_, the water molecules go into the sodium layers and form the hydration phases. Considering the full decomposition of generated sodium salts and the recrystallization of the degraded structures, once the Na_0.67_TmO_2_ is protonated or hydrated, high-temperature calcination is needed to recover the hydrated forms back to the original structure. In addition to the above structural transitions, the outer layer of Na_x_TmO_2_ particles decomposes into transition-metal oxides/hydroxides when exposed to air or immersed in water. As shown in Supplementary Fig. 20, after exposing Na_0.67_Cu_0.33_Mn_0.67_O_2_ at RH 93% + CO_2_ for 6 days, the Cu_2_O and NaHCO_3_ impurities can be observed in the XRD pattern, indicating that degradation is more or less an inevitable process at the surface of exposed Na_0.67_TmO_2_ compounds. Manthiram’s group also reported that the Ni-rich layered sodium oxide transforms into NiO and Na_2_CO_3_ in the surface^26^. In brief, the structural and chemical transitions coexist, rather than a single protonation/Tm-oxidation, hydration, and degradation mechanism.

### Evaluating the air-stability of Na_x_TmO_2_ electrodes

The choice and evaluation of air-stable Na_x_TmO_2_ electrodes have attracted enormous attention. It has been widely accepted that the Ni/Mn superlattice prevents the intercalation of H_2_O since P2-Na_0.67_Ni_0.33_Mn_0.67_O_2_ was found to be resistant to hydration in moisture^27^. However, our results demonstrate that Na_0.67_Ni_0.33_Mn_0.67_O_2_ is not as stable in moist air as it was expected, while other reported air-stable electrodes, such as Na_0.78_Cu_0.27_Zn_0.06_Mn_0.67_O_2_^32^, Na_0.67_Ni_0.22_Cu_0.11_Ti_0.11_Mn_0.56_O_2_^35^ and Na_7/9_Cu_2/9_Fe_1/9_Mn_2/3_O_2_^53^ do not have superlattice ordering in Tm layers. Therefore, the crucial factors, as well as the evaluation principles related to the air-stability of Na_x_TmO_2_ electrodes, have yet to be re-explored.

Therefore, we compared the air-stability of five layered oxides with different Mn valence-state and redox potential, e.g. Na_0.67_MnO_2_, Na_0.67_Al_0.1_Mn_0.9_O_2_, Na_0.67_Zn_0.1_Mn_0.9_O_2_, Na_0.67_Zn_0.2_Mn_0.8_O_2_ and Na_0.67_Ni_0.33_Mn_0.66_O_2_, at the atmosphere of dry CO_2_, RH 18%, RH 15% + CO_2_, and RH 93% + CO_2_. The XRD patterns and ICP-AES results of these pristine oxides are presented in Supplementary Fig. 21 and Table 5, respectively. In addition, the XRD patterns and the corresponding quantification of the hydration extents of the exposed samples are shown in Supplementary Fig. 22. According to the refinement results in Supplementary Fig. 22e, it is confirmed once again that more structural and chemical changes of Na_0.67_TmO_2_ occur with the increase of relative humidity and the presence of CO_2_. Moreover, it can be clearly observed that the air-stability of these investigated layered oxides follows the order of Na_0.67_Ni^2+^_0.33_Mn^4+^_0.67_O_2_ > Na_0.67_Zn^2+^_0.2_Mn^3.66+^_0.8_O_2_ > Na_0.67_Zn^2+^_0.1_Mn^3.47+^_0.9_O_2_ > Na_0.67_Al^3+^_0.1_Mn^3.37+^_0.9_O_2_ > Na_0.67_Mn^3.33+^O_2_, suggesting that the air-stability is closely related to the valence state of Mn ions or the redox potential of the initial charge process (Supplementary Fig. 23). To confirm this hypothesis, several electrodes were prepared and exposed to the atmosphere of RH 93% + CO_2_. As shown in Supplementary Fig. 24, although the valence state of Mn ions in Na_0.67_Co^3+^_0.67_Mn^4+^_0.33_O_2_, Na_0.67_Ni^2+^_0.17_Co^3+^_0.33_Mn^4+^_0.50_O_2_ and Na_0.67_Ni^2+^_0.17_Fe^3+^_0.33_Mn^4+^_0.50_O_2_ electrodes are Mn^4+^, all of these samples are hydrated after three days’ exposure in the atmosphere of RH 93% + CO_2_, indicating that the valence state of Mn ions is not the key parameter for the air-stability property. Therefore, Na_0.67_Li_0.20_Mn_0.80_O_2_^54^, Na_0.67_Mg_0.28_Mn_0.72_O_2_^55^ and Na_0.67_Cu_0.33_Mn_0.67_O_2_^56^ samples that have high initial charging potential were prepared and exposed to the same atmosphere. As shown in Supplementary Fig. 20 and 25, after the exposure for 3 or 6 days, no hydration peaks can be recognized, indicating that the Na_0.67_TmO_2_ components with higher redox potential in the first cycles exhibit better air-stability. Generally, for a Na_0.67_TmO_2_ material with higher electrochemical redox potential (equilibrium potential), it is more difficult to extract Na^+^ from the lattice chemically. Therefore, the potential of redox couples could be considered as one of the key principles to evaluate the air-stability of Na_0.67_TmO_2_ electrodes. Based on the above conclusion, we summarize the main redox couples in P2-Na_x_TmO_2_ electrodes (Fig. 7b), including V^3+/4+^^57^, Mn^3+/4+^^10^, Ru^4+/5+^^58^, Ir^4+/5+^^59^, Co^3+/4+^^7^, Cr^3+/4+^^60^, Fe^3+/4+^^61^, Ni^2+/4+^^8^, Cu^2+/3+^^56^, and O^2-/n-^^62^. The result suggests that the electrodes with redox couples of Ni^2+/4+^, Cu^2+/3+^, and O^2−/n−^ show much better air-stability than that with the redox couples of V^3+/4+^, Mn^3+/4+^, Co^3+/4+^, Fe^3+/4+^, etc., which is in good agreement with the previous articles^5,32,34,35,63^.

Besides chemical compositions, the crystallinity of Na_0.67_TmO_2_ also makes a great difference to their air-stability. Supplementary Fig. 26a,b show the weight loss and XRD evolutions during the preparation process of Na_0.67_MnO_2_, respectively. When the temperature increases to 480 °C, Na_2_CO_3_ begins to decompose and the β-MnO_2_ (JCPDF: 72–1984) starts to transform into the layered Na_0.67_MnO_2_. The XRD peaks of β-MnO_2_ disappear at ~540 °C and all of the Na_2_CO_3_ is decomposed at ~ 640 °C. We compared the air-stability of the Na_0.67_MnO_2_ samples at 640 °C, 900 °C, and the sample calcinated at 900 °C for 900 min, which exhibit increased crystallinity (Supplementary Fig. 26b). As shown in Supplementary Fig. 26c, after being exposed in RH 18% atmosphere for 3 days, the hydration degree follows the order of 640 °C > 900 °C > 900 °C 900 min samples, and clearly indicates that the samples with higher degree of crystallinity exhibit better air-stability.

## Discussion

In summary, a comprehensive investigation of the structural and chemical transformations of P2-Na_x_TmO_2_ in different ambient atmospheres has been carried out. Using advanced technique characterizations and systematic investigation, we clarified and rationalized these intertwined reaction processes and their determining factors. For example, the critical roles of relative humidity and CO_2_ in the air-stability of P2-Na_0.67_TmO_2_, the extraction of Na^+^ ions from Na_0.67_Ni_0.33_Mn_0.67_O_2_ when exposed to moist air or emerged in water, the influence of moisture-exposure on the electrochemical performances of Na_x_TmO_2_, the precise quantification of water contents in hydrated phases, and the significance of crystallinity on the air-stability of Na_x_TmO_2_, etc. Furthermore, based on the detailed mechanisms, including the Na extraction at the initial stage of moisture-exposure, the dominate Na^+^/H^+^ exchange charge-compensation mechanism, and the critical sodium content (*n*_*c*_), we proposed a general and in-depth picture on the chemical/structural evolutions of P2-Na_x_TmO_2_ during moisture-exposure. In addition, by extending the study to a variety of layered sodium-based oxides (Na_0.67_M_x_Mn_1-x_O_2_, with M = Ni, Zn, Fe, Al, Co, Li, Mg, Cu, etc.) we have also demonstrated that the redox potential properties in the first charge process could be used as an empirical rule for evaluating the air-stability of Na_x_TmO_2_ electrodes. Our results provide significant new clues for the design, synthesis, storage, and application of layered sodium cathodes, as well as other alkali-metal transition metal oxides.

## Methods

### Preparation of layered materials

The pristine Na_x_TmO_2_ cathodes have been synthesized by high-temperature solid-state reactions^12,23^. Stoichiometric amounts of raw materials, e.g. MnO_2_ (99.95%, Aladdin), ZnO (99.99%, Aladdin), NiO (99.99%, Aladdin), Al_2_O_3_ (99.99%, Aladdin), TiO_2_ (99.8%, Aladdin), CuO (99.9%, Aladdin), Li_2_CO_3_ (99.99%, Aladdin), and Na_2_CO_3_ (99.99%, Aladdin) were ball-milled with acetone solvent for 3.5 h at 500 rpm. Then dried at 120 °C overnight, pressed into pellets and heated at 900 °C for 15 h in air. After slowly cooled to 150 °C in the furnace, the pellets were transferred to an Ar-filled glove box immediately, ground and kept the final products in the Ar-filled glove box.

### Hydration tests

The saturated salt solutions could produce stable relative humidity in a closed system at a certain temperature^64,65^. To study the effect of different ambient atmospheres on the structural and chemical stability of Na_x_TmO_2_, the prepared Na_0.67_TmO_2_ oxides are put in a centrifuge tube and then aged on an airtight container under four different storage conditions at the constant temperature of 40 °C. They are dry CO_2_, controlled humidity of RH 18% (without CO_2_), RH 15% with CO_2_, and RH 93% with CO_2_. To maintain the ambience conditions of dry CO_2_, RH 15% with CO_2_ and RH 93% with CO_2_, the dry silica gel, saturated aqueous LiCl and NaHCO_3_ solutions are placed below the samples, respectively. After that, the containers are filled with CO_2_. The RH 18% (without CO_2_) atmosphere was obtained by placing the saturated NaOH solution at the bottom of the container and then filled with dry O_2_. The accurate RH is determined by a hygrometer, whose accuracy is ±1%. The elemental compositions of pristine and exposed samples were confirmed by inductively coupled plasma-atomic emission spectrometry (ICP-AES) analysis (IRIS Intrepid II XSP, Thermo Electron). Before ICP-AES and Time-of-flight secondary ion mass spectroscopy (TOF-SIMS) testing, we applied a scavenging process to remove the Na_2_CO_3_/NaHCO_3_ byproducts in the exposed samples. Specifically, the exposed samples were immersed and stirred in distilled water for 3 min, then centrifuged and dried at 80 °C overnight.

### Characterizations

The X-ray diffraction (XRD) patterns were obtained on a Rigaku Ultima IV diffractometer by a Persee instrument with Cu Kα radiation (*λ* = 1.5406 Å). The patterns were refined by the General Structure Analysis System (GSAS) software^44^. The morphologies of samples were characterized by scanning electron microscopy (SEM, Hitachi S-4800). The EDS elemental mapping experiments were conducted using FEI Talos-F200s TEM instrument. The ^23^Na ss-NMR experiments were acquired on a Bruker AVANCE III 400 MHz spectrometer using a double resonance 1.3 mm MAS probe spinning at frequencies of up to 55 kHz with a Hahn-echo pulse sequence (i.e. 90^o^ pulse – τ – 180^o^ pulse – τ, where τ is set to a multiple of rotor periods). The 90^o^ pulse length of 1.2 μs and a recycle delay of 2 ms were used. The ^23^Na shifts were referenced to 1 M NaCl aqueous solution (0 ppm). For ^1^H, the 2.5 mm MAS probe was used spinning at 25 kHz to record the ^1^H spectra with the rotor-synchronized Hahn Echo sequence and a cycle delay of 2 s and a 90^o^ pulse length of 4 μs. No window functions were added and the chemical shift of ^1^H was referenced against adamantane (1.87 ppm). By using 2.5 mm MAS probe with a MAS rate of 25 kHz, the rotational echo double resonance (REDOR) NMR sequence (i.e. the rotor-synchronized Hahn echo is applied on ^23^Na, during τ periods the ^23^Na-^1^H dipolar coupling is recovered by 180^o 1^H pulses) is applied to confirm the presence of H^+^ in the exposed materials. Geometry-independent information about the ^23^Na-^1^H dipole couplings can be conveniently obtained from the plot of signal attenuation (1-scale) versus the dipolar evolution time (2tau). In all MAS NMR measurements, the variable temperature (VT) gas temperature was set to 315 K. The in situ XRD experiments were performed in air and on a BrukerD8 Discover diffractometer equipped with a Cu Kα radiation. Infrared spectra were recorded on a Nicolet is50 FT-IR (Thermo Fisher Scientific Inc., Madison, USA) spectrometer. X-ray absorption spectroscopy (XAS) data were acquired in the transmission mode at the BL14W1 beamline of the Shanghai synchrotron radiation facility (SSRF) at room temperature, and the incident beam was monochromatized by a Si (111) double-crystal monochromator. TOF-SIMS were performed on a TOF-SIMS5 spectrometer (ION TOF GmbH). The depth profiling and high-resolution mapping were conducted at the high current mode and burst alignment mode, respectively, with a pulsed Bi_1_^+^ ion beam (30 keV). The sputtering was conducted by a 500 eV Cs^+^ ion beam with the area of 100 μm × 100 μm, and the analyzed areas for depth profiling and high-resolution mapping are typically 100 μm × 100 μm and 50 μm × 50 μm with the pixel of 128 × 128.

### Electrochemical tests

The electrodes are composed of 80 wt% of active materials, 10 wt% of polyvinylidene fluoride (PVDF) and 10 wt% acetylene carbon black. The mass loading of active materials is 2.5–3 mg cm^−1^. The cleaned aluminum foil was used as current collector. The electrochemical performances were tested in 2025 coin cells which assembled in an Ar-filled glove box, using 1 M NaPF_6_ in Propylene carbonate (PC, 98 vol%) and Fluoroethylene carbonate (FEC, 2 vol%) as the electrolyte, Whatman glass fiber filter as the separator, and sodium metal as the counter electrode. The galvanostatic charge-discharge processes were conducted on multichannel battery tester (Neware, CT-4008-5V10mA-164). The electrochemical impedance spectroscopy (EIS) was conducted by a four-channel multifunctional electrochemical workstation (Versa STAT MC. America), at the cell voltage of 4.0 V and with the frequency range of 100 kHz to 0.01 Hz.

### DFT calculation

The DFT calculations were performed on VASP (Vienna ab initio Simulation Package)^66^ and the exchange-correlation interactions of electron were described with spin-polarized generalized gradient approximation (GGA) and parameterized by PBE formula^67^. The projector-augmented wave approach and GGA + U method were used to evaluate the electron-ion interactions and the localization of the d electrons of the TM ions, respectively^68,69^. The U value of Mn set to 3.9 eV and the wave functions were expended by plane wave with a kinetic energy cut-off of 520 eV. The Monkhorst–Pack scheme^70^ was used for the integration in the irreducible Brillouin zone with a k-point mesh resolution of 2π × 0.025 Å^−1^. The lattice parameters and atomic coordinates were fully relaxed, and the final forces on all atoms were <0.01 eV Å^−1^. For the DFT calculations, a 3 × 3 × 1 supercell, which contains 12 Na, 36 O, and 18 Mn has been adopted. Sodium contents of *x* = 0.667, 0.556, 0.500, 0.444 and 0.389 in Na_x_MnO_2_ correspond to 12, 10, 9, 8 and 7 Na ions in the supercell, and the number of different structures for each Na contents is $C_{12}^0$, $C_{12}^2$, $C_{12}^3$, $C_{12}^4$, and $C_{12}^5$, respectively. Therefore, 1574 kinds of structures should be considered based on the enumeration method. Such computation load is however too heavy to be carried out and 9 of them have been calculated. Specifically, we considered two different sodium extraction models for the five different Na contents. The number of calculated structures for *x* = 0.667, 0.556, 0.500, 0.444 and 0.389 in Na_x_MnO_2_ are 1, 2, 2, 2, 2, respectively, as shown in Fig. 4f (page 11 in the manuscript). Moreover, the residual Na sites were chosen randomly and then one water molecule was put into an empty Na site also randomly.

## Supplementary information

Supplementary Information

## Data Availability

All relevant data that support the findings of this study are presented in the manuscript and supplementary information file. Source data are available from the corresponding author on request.
