# Supplementary material for: The stability of P2-layered sodium transition metal oxides in ambient atmospheres
Source: Nat Commun. 2020 Jul 15;11:3544. doi: 10.1038/s41467-020-17290-6 (PMC7363866; doi:10.1038/s41467-020-17290-6)
Supplement: Supplementary file 1 — Supplementary Information [file 41467_2020_17290_MOESM1_ESM.pdf]

Supplementary Information for

The stability of P2-layered sodium transition metal oxides in ambient atmospheres

Wenhua Zuo[a], Jimin Qiu[a], Xiangsi Liu[a], Fucheng Ren[b], Haodong Liu[c], Huajin He[a], Chong Luo[d], Jialin Li[a], Gregorio F. Ortiz[a,e], Huanan Duan[f], Jinping Liu[g,*], Ming-Sheng Wang[d], Yangxing Li[h], Riqiang Fu[i], Yong Yang[a,b,*]

[a] *State Key Laboratory for Physical Chemistry of Solid Surfaces, and Department of Chemistry, College of Chemistry and Chemical Engineering, Xiamen University, Xiamen 361005, People's Republic of China*

[b] *School of Energy Research, Xiamen University, Xiamen 361005, People's Republic of China*

[c] *Department of NanoEngineering, University of California San Diego, Lga Jolla, California 92093, USA*

[d] *Department of Materials Science and Engineering, College of Materials, Xiamen University, Xiamen, Fujian 361005, People's Republic of China*

[e] *Departamento de Química Inorgánica e Ingeniería Química, Instituto Universitario de Investigación en Química Fina y Nanoquímica, Universidad de Córdoba, Campus de Rabanales, Edificio Marie Curie, E-14071, Córdoba, Spain*

[f] *State Key Laboratory of Metal Matrix Composites, School of Materials Science and Engineering, Shanghai Jiao Tong University, Shanghai 200240, P. R. China*

[g] *School of Chemistry, Chemical Engineering and Life Science and State Key Laboratory of Advanced Technology for Materials Synthesis and Processing, Wuhan University of Technology, Wuhan, Hubei 430070, People's Republic of China*

[h] *4135 Belle Meade Circle, Belmont, NC 28012, USA*

[i] *National High Magnetic Field Laboratory, 1800 E. Paul Dirac Drive, Tallahassee, FL 32310, USA*

Corresponding author: * liujp@whut.edu.cn (J. Liu) * yyang@xmu.edu.cn (Y. Yang)

Supplementary Figures

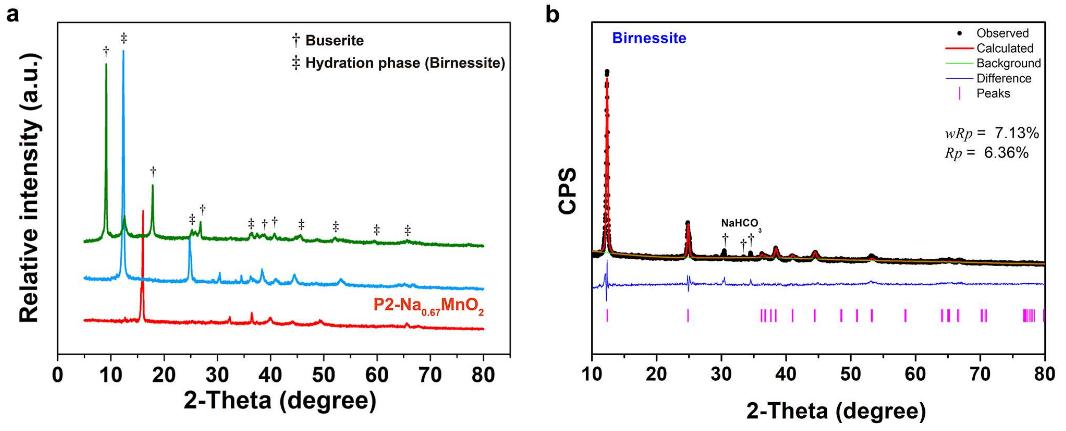

Supplementary Figure 1. The XRD patterns for hydration phases. (a) The XRD patterns of $Na_{0.67}MnO_2$, birnessite, and buserite phases. (b) The Rietveld refinement patterns of birnessite phase.

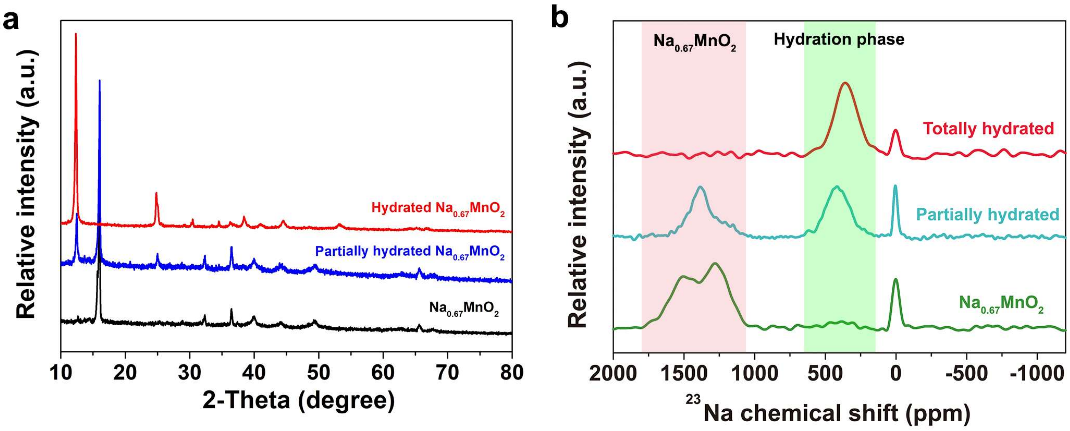

Supplementary Figure 2. ^{23}Na MAS NMR spectra of birnessite phase. The (a) XRD patterns and (b) ^{23}Na MAS NMR spectra of pristine $Na_{0.67}MnO_2$, partially hydrated $Na_{0.67}MnO_2$ (pristine + birnessite phases), and totally hydrated $Na_{0.67}MnO_2$ (birnessite phase) materials.

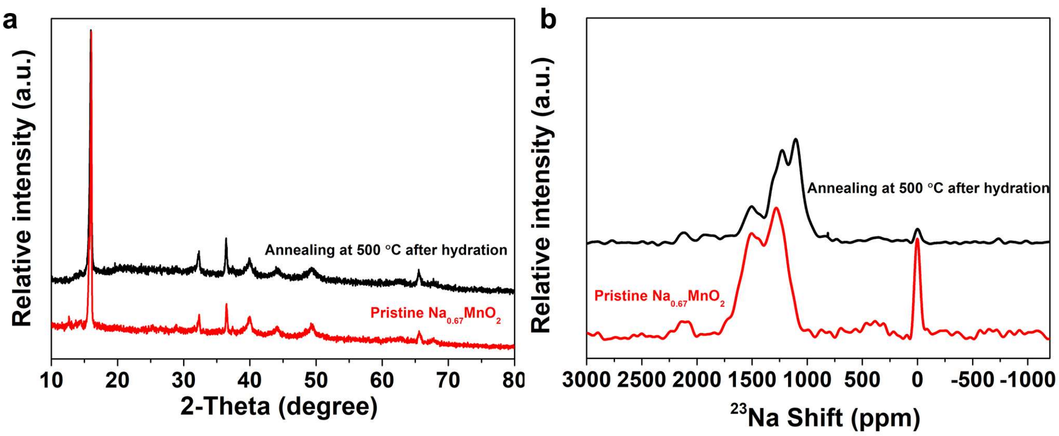

Supplementary Figure 3. XRD pattern and ^{23}Na ss-NMR spectrum of dehydrated oxide. (a) XRD pattern and (b) ^{23}Na ss-NMR spectrum (MAS rate: 55 kHz) of the totally hydrated $Na_{0.67}MnO_2$ sample after annealing at 500 ℃.

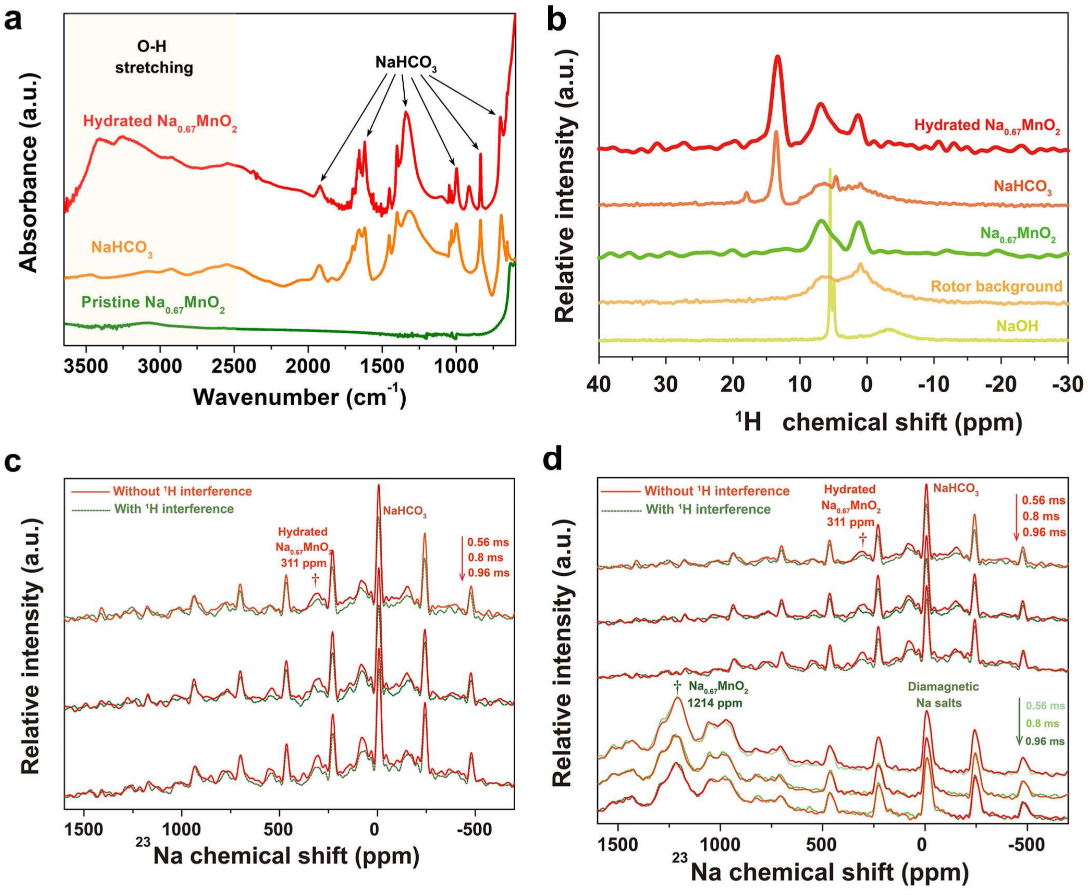

Supplementary Figure 4. FTIR, ^{1}H MAS NMR and ^{23}Na{^{1}H} REDOR MAS NMR spectra of birnessite phase. (a) The FTIR spectra of pristine and hydrated $Na_{0.67}MnO_2$ samples, in which the new peaks located at 800-2000 cm^{-1} corresponds to $NaHCO_3$. (b) ^{1}H MAS NMR spectra (MAS rate: 25 kHz) and c) ^{23}Na{^{1}H} REDOR-dephased ss-NMR spectra (MAS rate: 25 kHz) of pristine and totally hydrated $Na_{0.67}MnO_2$ samples. (d) Enlarged ^{23}Na{^{1}H} REDOR-dephased ss-NMR spectra of totally hydrated $Na_{0.67}MnO_2$ samples.

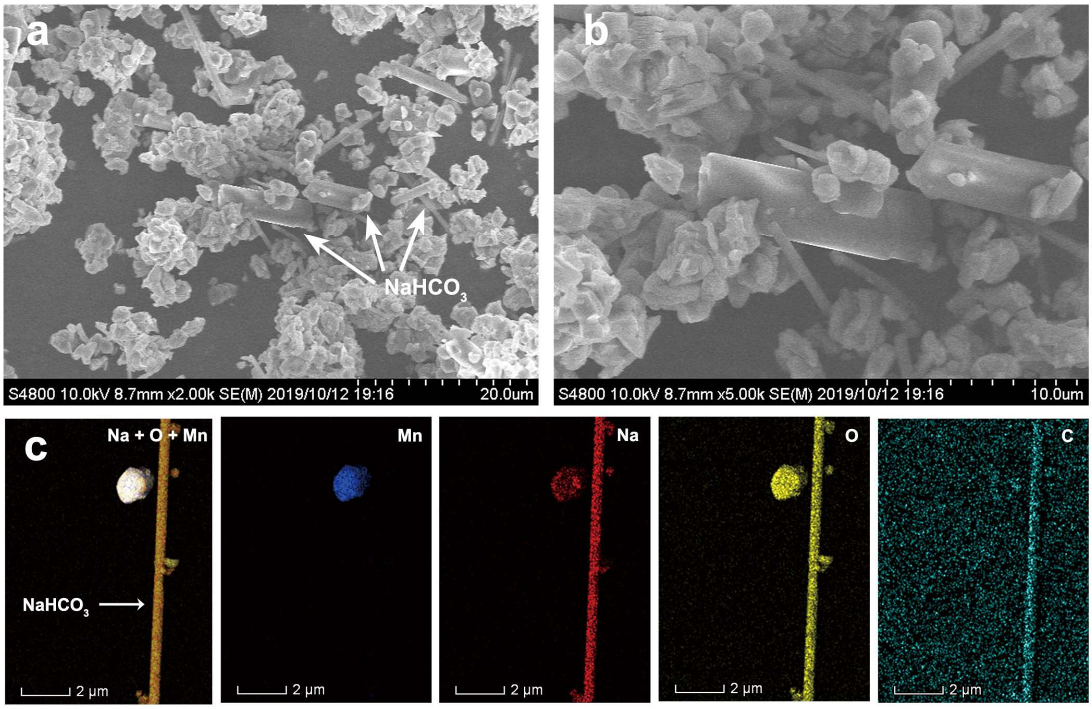

Supplementary Figure 5. $NaHCO_3$ crystals. (a-b) The SEM images of exposed $Na_{0.67}MnO_2$ to RH 93% + CO_2 for 3 days. (c) The EDS mapping results, which confirm that the crystals with regular shapes are $NaHCO_3$.

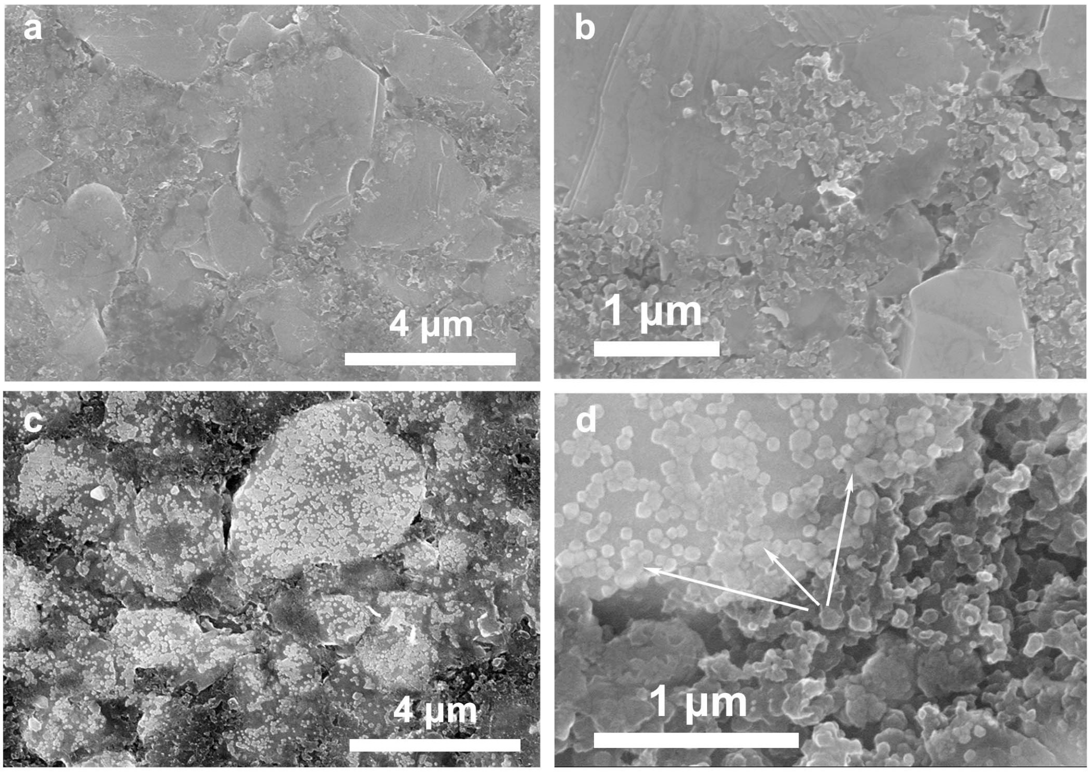

Supplementary Figure 6. Formation of sodium salts in air-exposed electrode. The SEM images for (a, b) as-prepared and (c, d) air-exposed (3 days) $Na_{0.67}MnO_2$ electrodes.

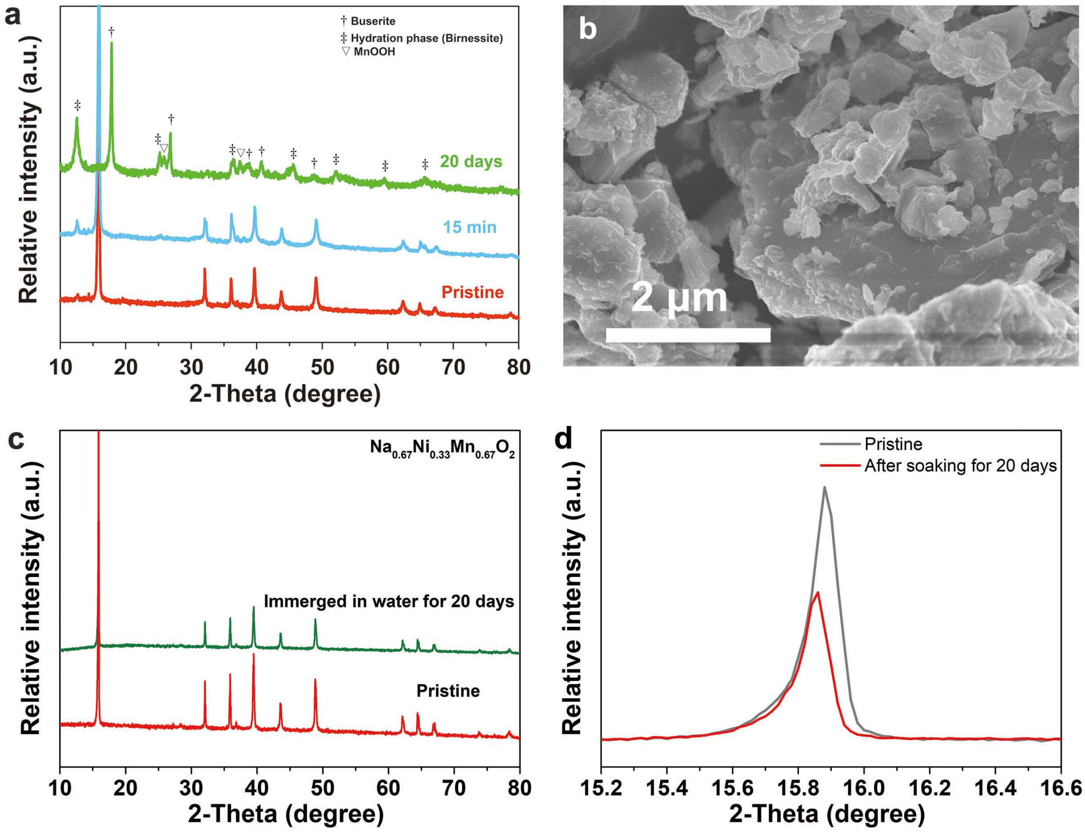

Supplementary Figure 7. Immersing in water. (a) The XRD evolutions of $Na_{0.67}MnO_2$ materials immersed in water for different times. (b) The SEM images of $Na_{0.67}MnO_2$ materials after immersing in water for 20 days. (c) The XRD patterns of $Na_{0.67}Ni_{0.33}Mn_{0.67}O_2$ immersed in water for 20 days. (d) The (002) XRD peak of pristine $Na_{0.67}Ni_{0.33}Mn_{0.67}O_2$ and $Na_{0.67}Ni_{0.33}Mn_{0.67}O_2$ samples after soaking in water for 20 days.

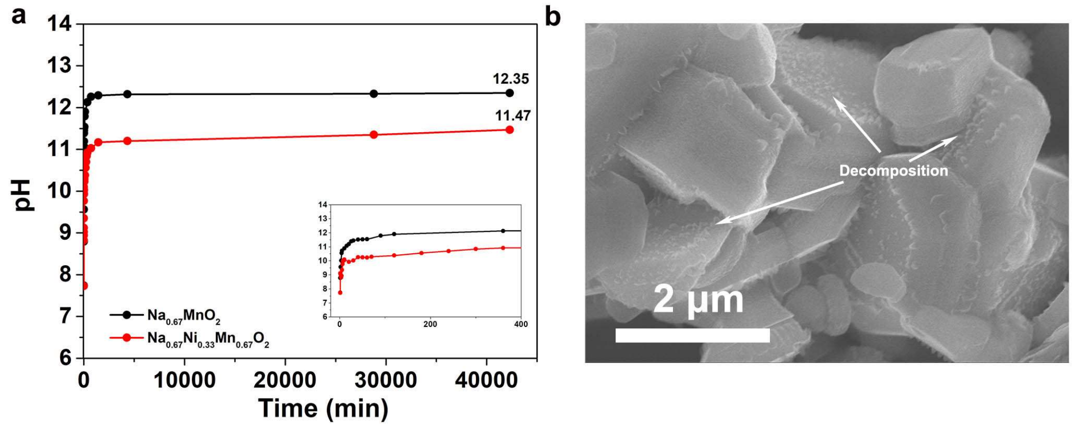

Supplementary Figure 8. Na^{+} loss in water-immersed $Na_{0.67}Ni_{0.33}Mn_{0.67}O_2$ sample. (a) The pH evolutions of the aqueous solutions after immersing $Na_{0.67}MnO_2$ and $Na_{0.67}Ni_{0.33}Mn_{0.67}O_2$ samples. (b) The SEM images of $Na_{0.67}Ni_{0.33}Mn_{0.67}O_2$ materials after immersing in water for 20 days.

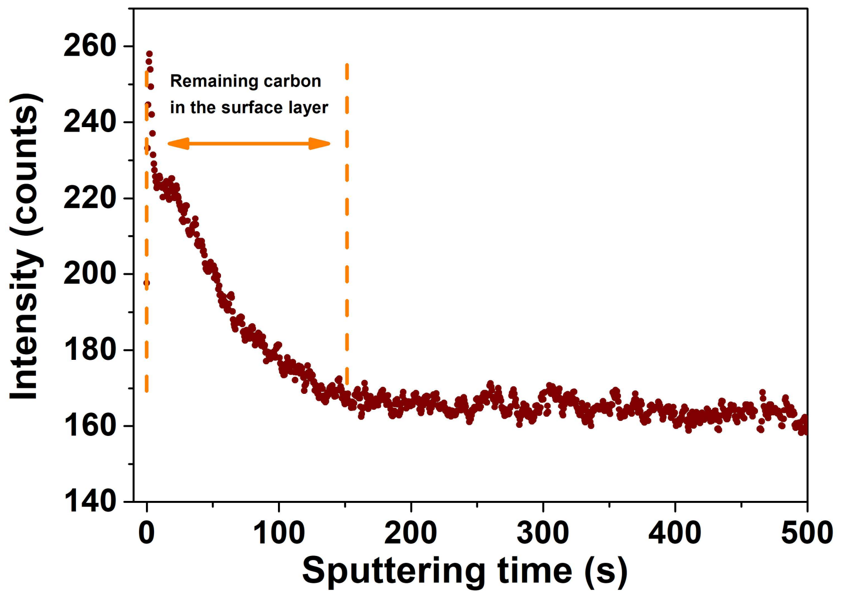

Supplementary Figure 9. Enlarged TOF-SIMS spectra. The enlarged TOF-SIMS spectra of C_2HO^- ions over 500 s Cs^+ sputtering on the hydrated $Na_{0.67}MnO_2$ samples after $NaHCO_3$ scavenging.

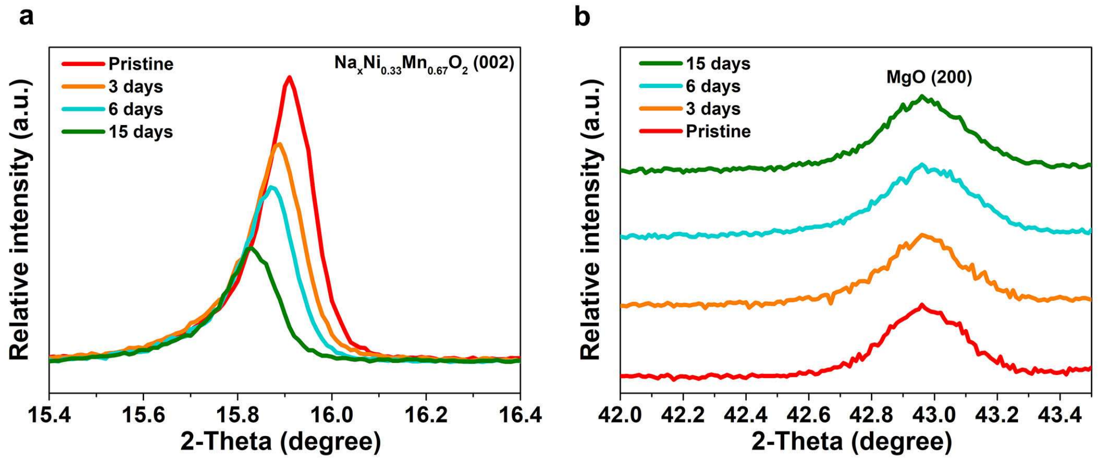

Supplementary Figure 10. Sodium extraction in moisture exposed $Na_{0.67}Ni_{0.33}Mn_{0.67}O_2$. The XRD patterns ranging from (a) 15.4º-16.4º and (b) 42.0º-43.5º of exposed $Na_{0.67}Ni_{0.33}Mn_{0.67}O_2$ (with MgO as internal indicator) in RH 93% + CO_2 with different exposure times. The intensity decreases of (002) peak and the shift of (002) peak to lower 2-Theta degree suggest the expansion of the sodium layers and the continual extraction of Na^+ in $Na_{0.67}Ni_{0.33}Mn_{0.67}O_2$ upon exposure. Moreover, the (200) peaks of MgO indicator in the three exposed samples are all located at 42.96º, indicating that the shift of (002) peaks of $Na_{0.67}Ni_{0.33}Mn_{0.67}O_2$ component is reliable. The mass ratio between exposed $Na_{0.67}Ni_{0.33}Mn_{0.67}O_2$ and MgO is 1:1.5.

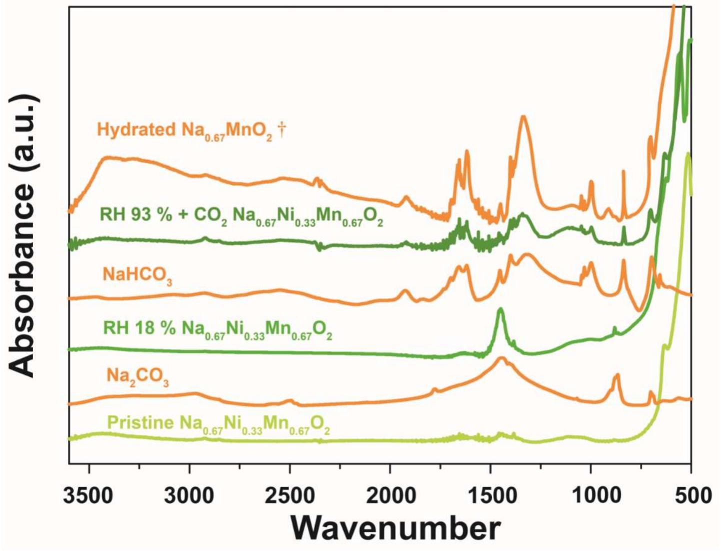

Supplementary Figure 11. FTIR spectra of exposed $Na_{0.67}Ni_{0.33}Mn_{0.67}O_2$. The FTIR spectra of exposed $Na_{0.67}Ni_{0.33}Mn_{0.67}O_2$ at RH 93% + CO_2 and RH 18% for 15 days, with Na_2CO_3 and $NaHCO_3$ spectra for comparison. New FTIR peaks correspond to Na_2CO_3 and $NaHCO_3$ can be observed in $Na_{0.67}Ni_{0.33}Mn_{0.67}O_2$ samples exposed at RH 18% and RH 93% + CO_2 atmosphere, respectively, which are important to obtain the composition of hydrated samples, as we will discuss in the following sections.

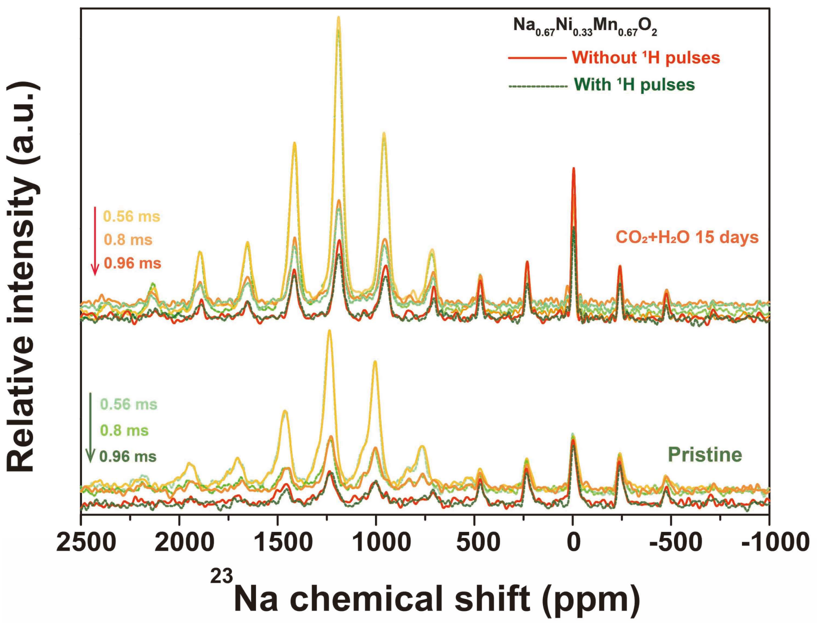

Supplementary Figure 12. $^{23}Na\{^1H\}$ REDOR MAS NMR spectra of air-exposed $Na_{0.67}Ni_{0.33}Mn_{0.67}O_2$. The $^{23}Na\{^1H\}$ REDOR-dephased ^{23}Na ss-NMR spectra (MAS rate: 25 kHz) ranging from -1000 to 2500 ppm of the exposed $Na_{0.67}Ni_{0.33}Mn_{0.67}O_2$ sample at RH 93%+CO_2 for 15 days.

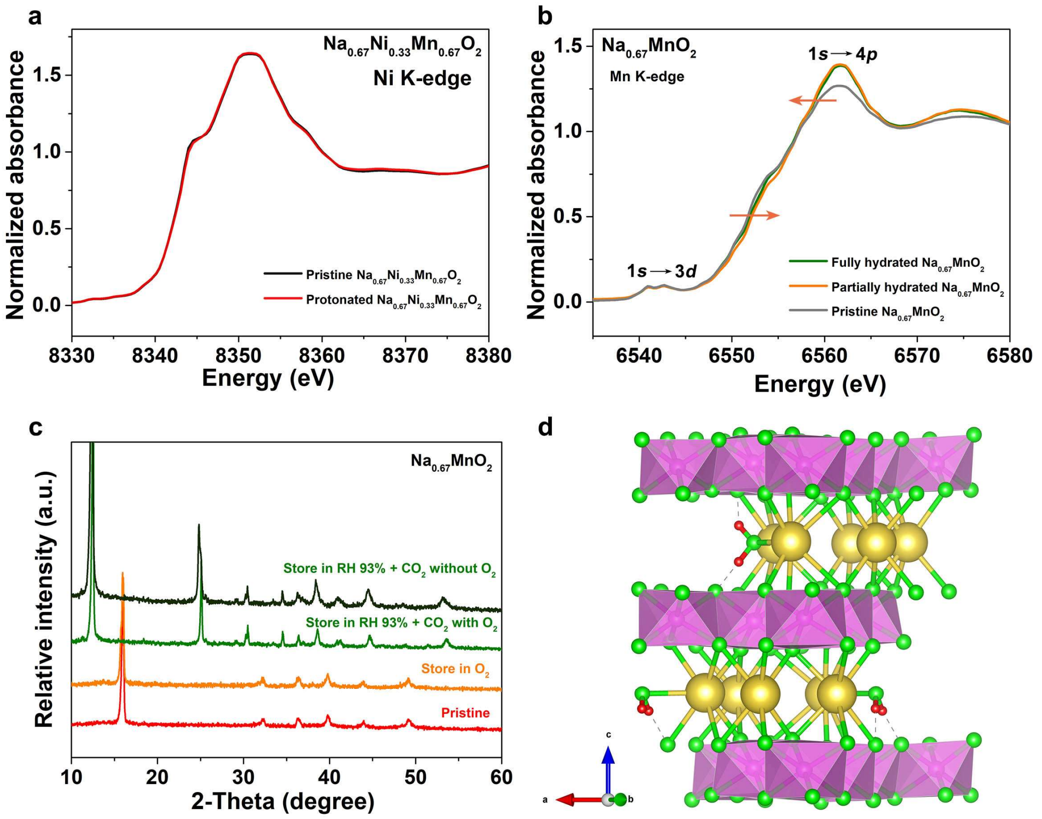

Supplementary Figure 13. The structural and chemical evolution mechanisms upon air-exposure. (a) The K-edge XAS of Ni for pristine and exposed $Na_{0.67}Ni_{0.33}Mn_{0.67}O_2$ sample. (b) The K-edge XAS of Mn for pristine, partially hydrated and totally hydrated $Na_{0.67}MnO_2$ samples. (c) The XRD patterns for $Na_{0.67}MnO_2$ samples stored in three different atmospheres for 3 days. (d) The sodium extraction models of double-layer model for calculating the hydration energies.

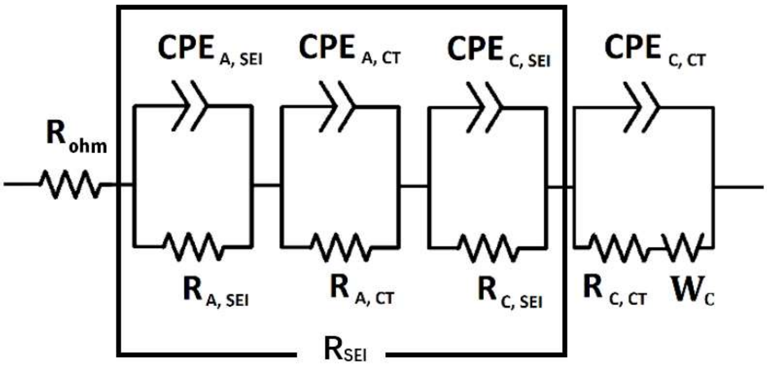

Supplementary Figure 14. Equivalent circuit. The equivalent circuit used for EIS analysis in this study. The impedance of electrodes can be attributed to Ohmic resistance (R_{ohm}), charge transfer resistance of the anode ($R_{A, CT}$) and the cathode ($R_{C, CT}$), the surface resistance of the anode ($R_{A, SEI}$) and cathode ($R_{C, SEI}$), and Warburg resistance. Moreover, because the $R_{A, SEI,}$ $R_{A, CT}$ and $R_{C, SEI}$ locate at the high-frequency region, they often coincide and form a semi-circle in the high-frequency region of the Nyquist spectra.[1]

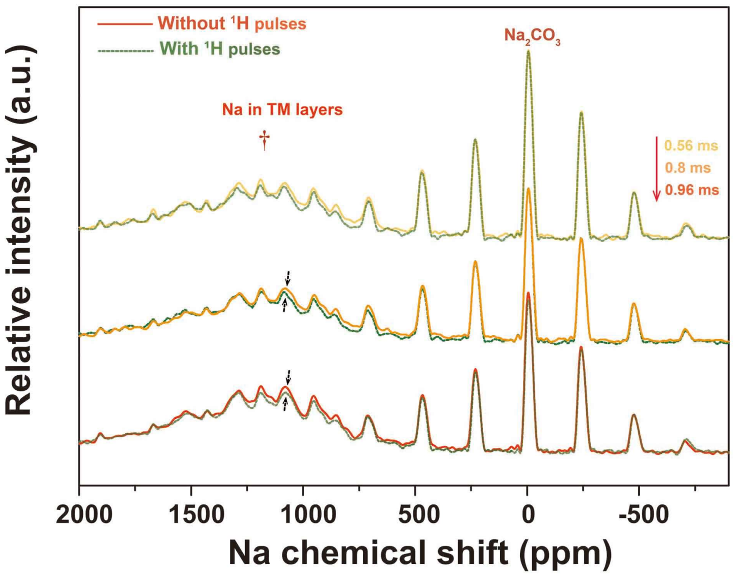

Supplementary Figure 15. $^{23}Na\{^1H\}$ REDOR spectra of 150 °C annealed samples. The $^{23}Na\{^1H\}$ REDOR-dephased ^{23}Na ss-NMR spectra (MAS rate: 25 kHz) of the hydrated $Na_{0.67-x-z}H_xMnO_2$ sample after annealing at 150 °C for 3 minutes.

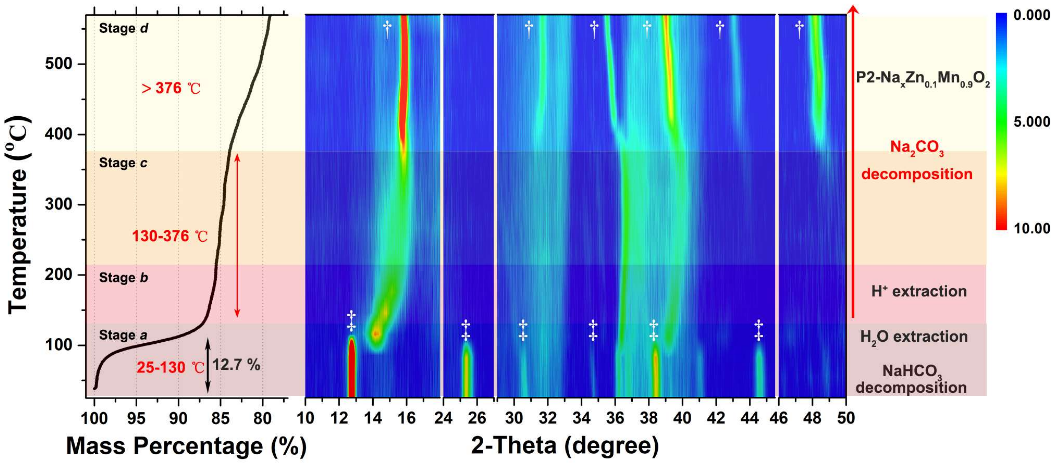

Supplementary Figure 16. Temperature-resolved *in-situ* XRD patterns. Temperature-resolved *in-situ* XRD patterns for the hydrated $Na_{0.67}Zn_{0.1}Mn_{0.9}O_2$ powder ranging from 25-570 ℃.

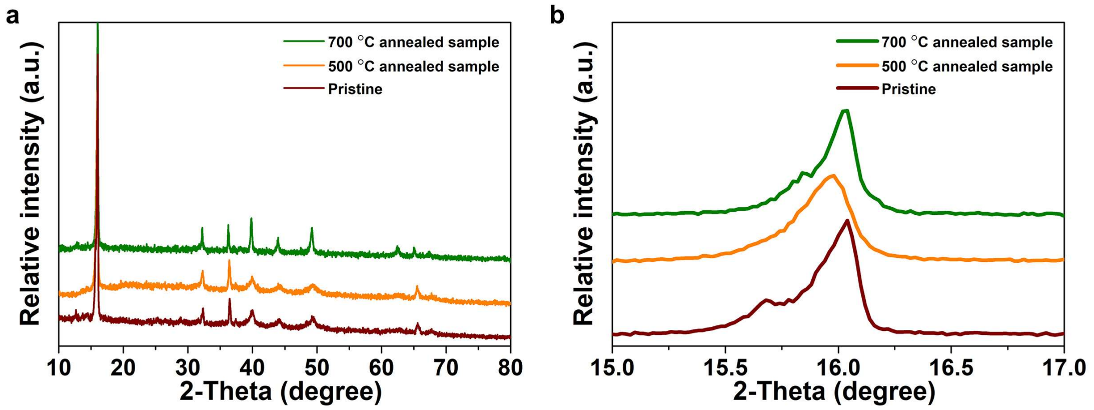

Supplementary Figure 17. Verifying the healing effect of high temperature annealing. The XRD patterns of pristine $Na_{0.67}MnO_2$, 500 °C-annealed hydration sample, and 700 °C-annealed hydration sample ranging from (a) 10^0 - 80^0 and (b) 15^0 - 17^0.

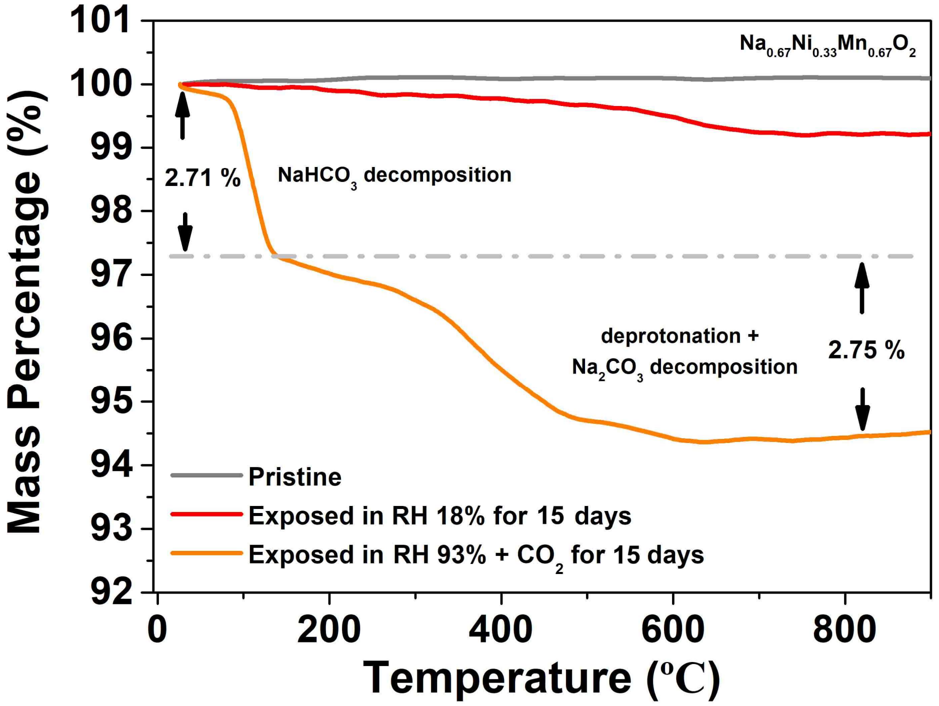

Supplementary Figure 18. TGA curves of $Na_{0.67}Ni_{0.33}Mn_{0.67}O_2$ samples. The TGA results of pristine $Na_{0.67}Ni_{0.33}Mn_{0.67}O_2$ sample, $Na_{0.67}Ni_{0.33}Mn_{0.67}O_2$ sample exposed in RH 18% for 15 days and $Na_{0.67}Ni_{0.33}Mn_{0.67}O_2$ sample exposed in RH 93% + CO_2 for 15 days.

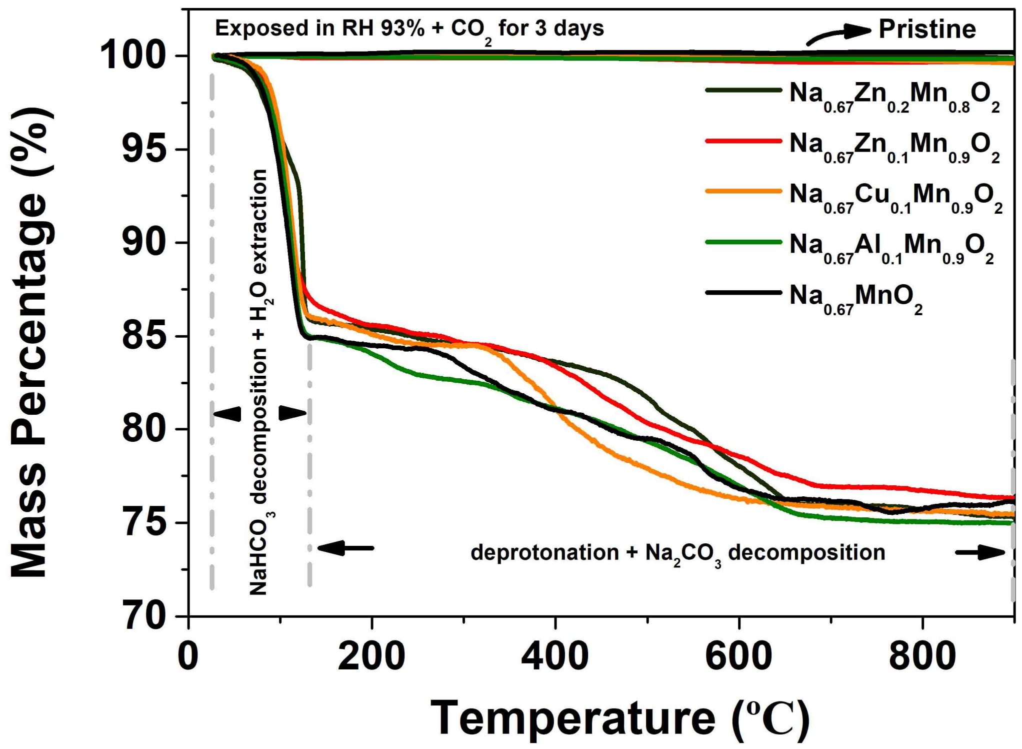

Supplementary Figure 19. TGA curves of hydration phases. The TGA results of pristine and hydrated $Na_{0.67}MnO_2$, $Na_{0.67}Al_{0.1}Mn_{0.9}O_2$, $Na_{0.67}Cu_{0.1}Mn_{0.9}O_2$, $Na_{0.67}Zn_{0.1}Mn_{0.9}O_2$, $Na_{0.67}Zn_{0.2}Mn_{0.8}O_2$ samples.

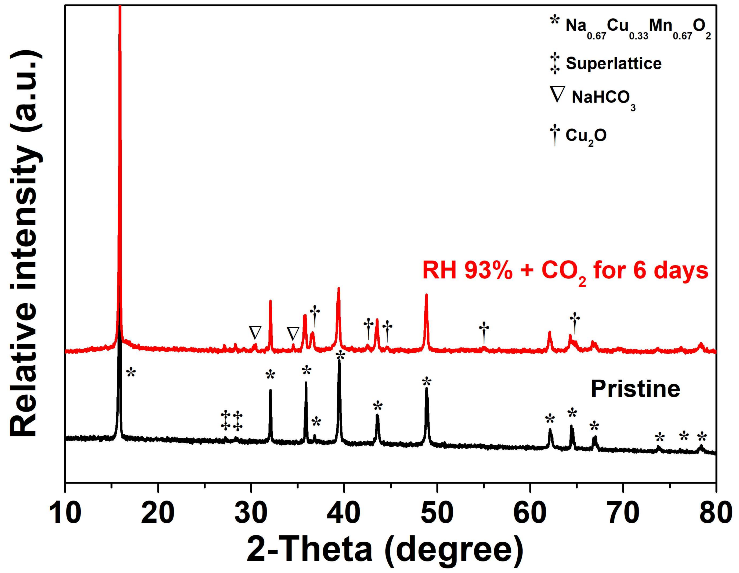

Supplementary Figure 20. Surface degradation. The comparison of XRD patterns of pristine and exposed $Na_{0.67}Cu_{0.33}Mn_{0.67}O_2$ samples. The sample was exposed in RH 93% + CO_2 for 6 days.

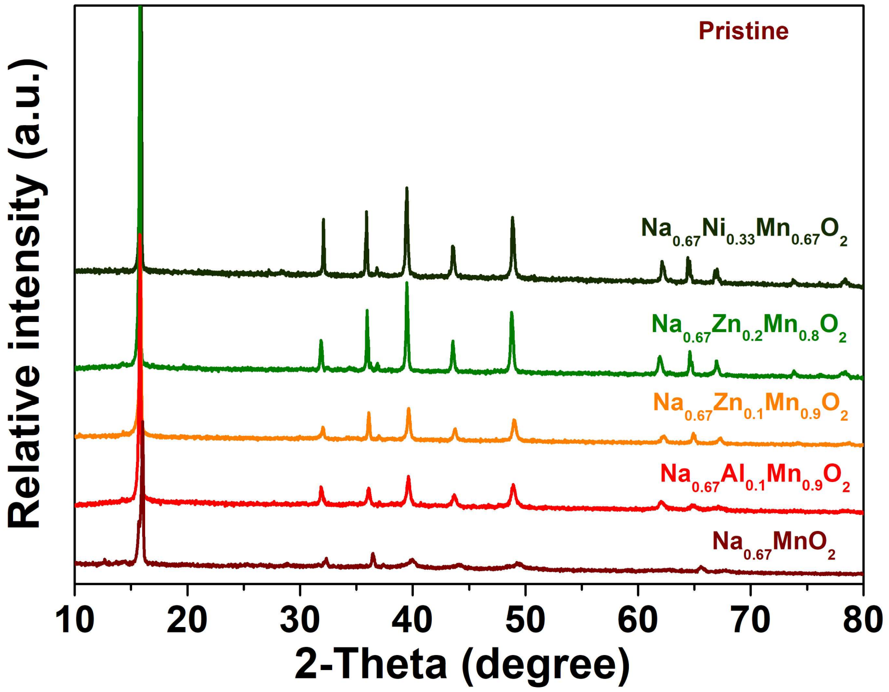

Supplementary Figure 21. Pristine compounds. The XRD patterns of pristine $Na_{0.67}MnO_2$, $Na_{0.67}Al_{0.1}Mn_{0.9}O_2$, $Na_{0.67}Zn_{0.1}Mn_{0.9}O_2$, $Na_{0.67}Zn_{0.2}Mn_{0.8}O_2$ and $Na_{0.67}Ni_{0.33}Mn_{0.66}O_2$. All of the diffraction peaks can be indexed to P2-type structure.[2]

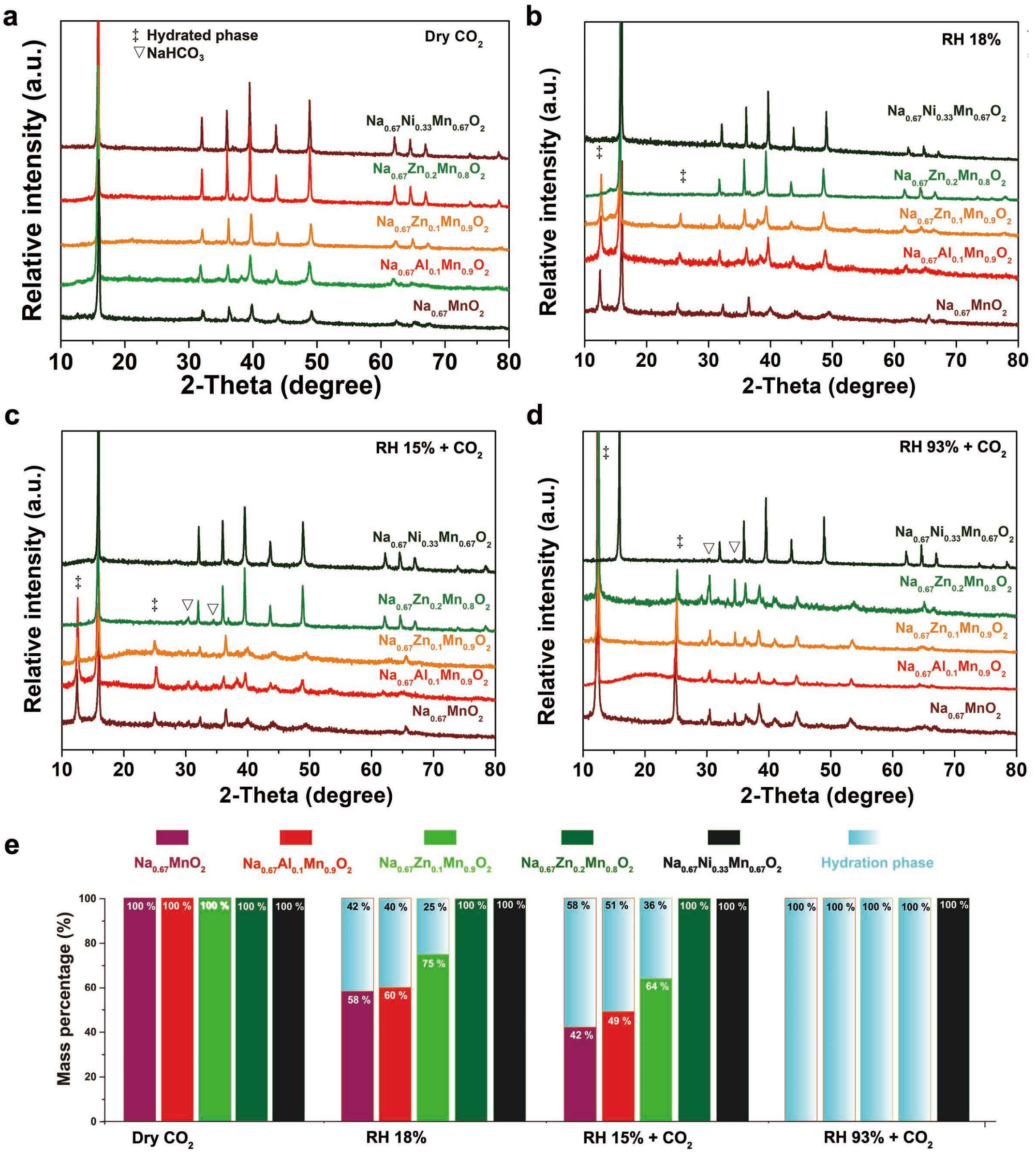

Supplementary Figure 22. Air-stability of different layered oxides. (a-d) The XRD patterns of $Na_{0.67}TmO_2$ oxides after exposing at various atmospheres for 3 days. (e) The Rietveld refinement results of exposed samples with a two-phase model.

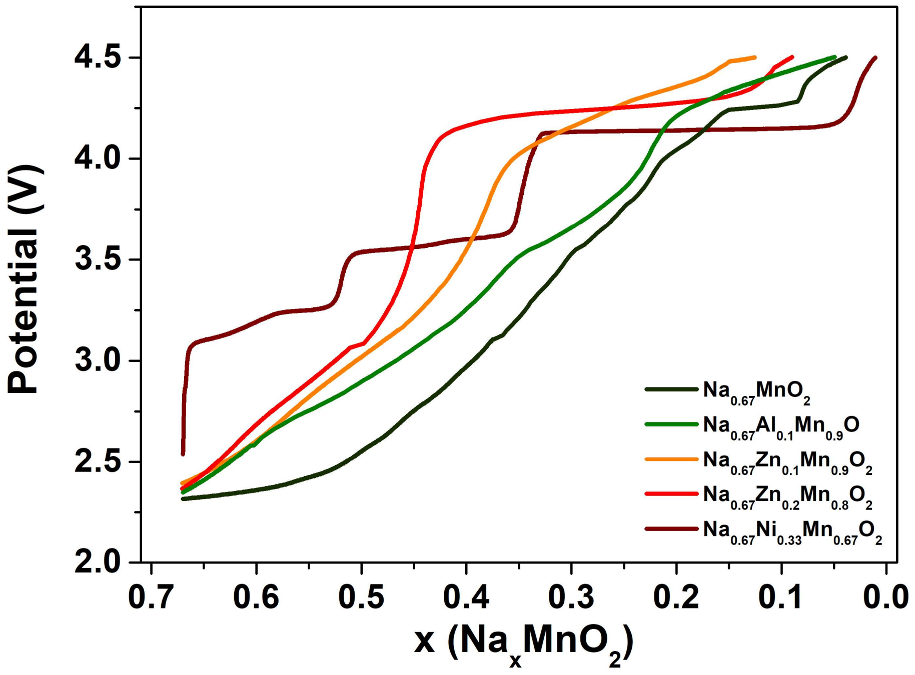

Supplementary Figure 23. Initial charge-discharge curves. The initial charge-discharge curves of $Na_{0.67}MnO_2$, $Na_{0.67}Al_{0.1}Mn_{0.9}O_2$, $Na_{0.67}Zn_{0.1}Mn_{0.9}O_2$, $Na_{0.67}Zn_{0.2}Mn_{0.8}O_2$ and $Na_{0.67}Ni_{0.33}Mn_{0.67}O_2$ electrodes.

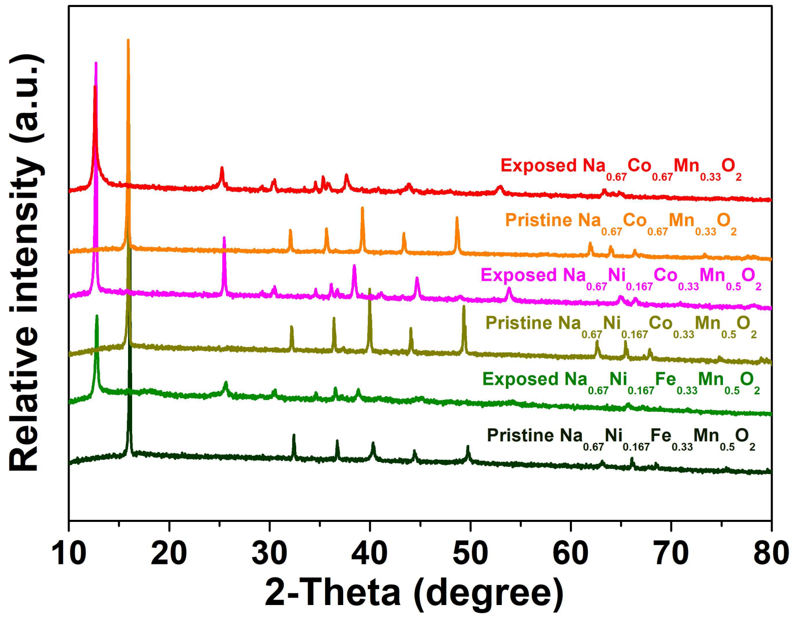

Supplementary Figure 24. Verification of evaluation rule. The XRD patterns of pristine and exposed $Na_{0.67}Co_{0.67}Mn_{0.33}O_2$, $Na_{0.67}Ni_{0.167}Co_{0.33}Mn_{0.5}O_2$ and $Na_{0.67}Ni_{0.167}Fe_{0.33}Mn_{0.5}O_2$ samples. The exposed samples were placed in RH 93% + CO_2 atmosphere for 3 days.

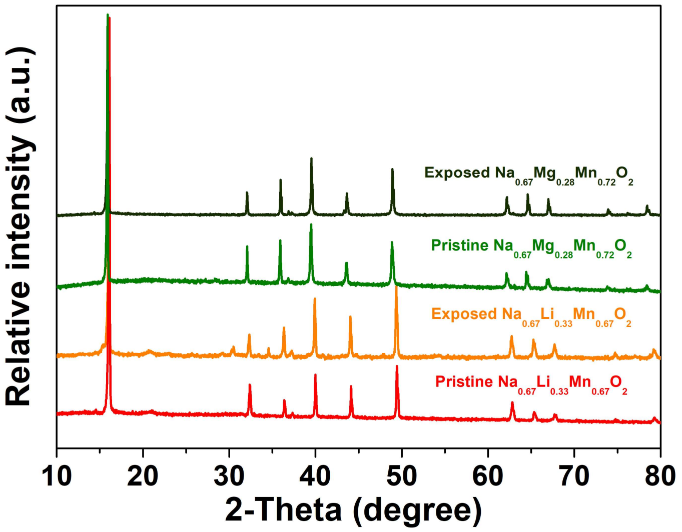

Supplementary Figure 25. Verification of evaluation rule. The XRD patterns of pristine and exposed $Na_{0.67}Li_{0.33}Mn_{0.67}O_2$ and $Na_{0.67}Mg_{0.28}Mn_{0.72}O_2$ samples. The exposed samples were exposed in RH 93% + CO_2 for 3 days.

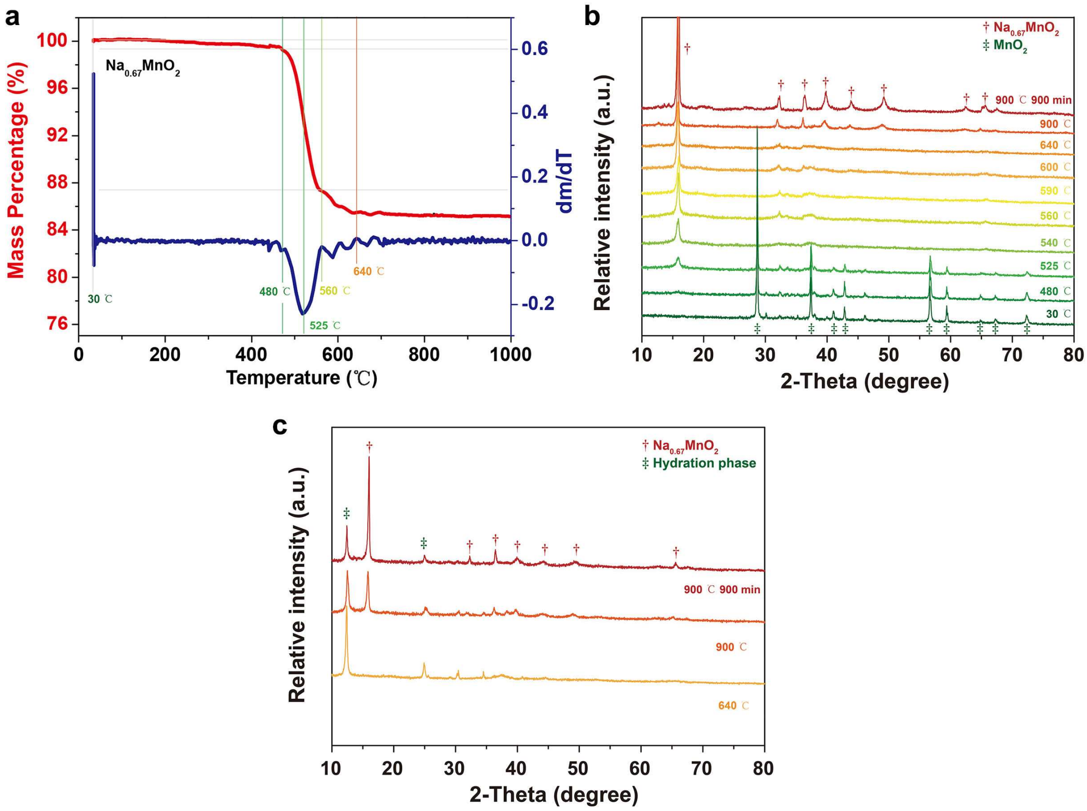

Supplementary Figure 26. The influence of crystallinity on air-stability. (a) The weight loss and (b) the XRD evolutions during the preparation process of $Na_{0.67}MnO_2$. (c) the XRD patterns of the $Na_{0.67}MnO_2$ samples at different calcination stages after the exposure in RH 18% for 3 days.

Supplementary Tables

Supplementary Table 1. Crystal parameters of birnessite phase. Refined crystallographic parameters obtained by Rietveld refinement of birnessite Na_xMnO_2.

Atom	X	Y	Z	Occupancy	B_{iso}	*a* (Å)	*c* (Å)	*V* (Å^3)	*wRp*
Na_f (1)	0.0	0.0	1/4	0.11	0.15				
Na_e (2)	1/3	2/3	3/4	0.19	0.80				
Mn	0.0	0.0	0.0	1	0.04	2.8984	14.2572	104.455	7.43 %
O	2/3	1/3	0.072	1	0.03				
O (H_2O)	1/3	2/3	3/4	0.45	0.025				

Attention: The O (H_2O) site reported by Dahn'group[3] is (1/3, 2/3, 1/4). In this study, a better fit was obtained by using the O (H_2O) site of (1/3, 2/3, 3/4) (wRp: 7.43 %) than (1/3, 2/3, 1/4) (wRp: 8.91 %). Moreover, our DFT calculation also confirms that the O ions of inserted H_2O locate in the Na^+ vacancy sites.

Supplementary Table 2. ICP-AES results. ICP-AES results for prepared $Na_{0.67}MnO_2$ and $Na_{0.67}Ni_{0.33}Mn_{0.67}O_2$ oxides.

Oxides	Tm ions	Atomic Ratios
$Na_{0.67}MnO_2$	$n_{Na} : n_{Mn}$	0.675 : 1.000
$Na_{0.67}Ni_{0.33}Mn_{0.67}O_2$	$n_{Na} : n_{Ni} : n_{Mn}$	0.665 : 0.341 : 0.659

Supplementary Table 3. EIS analysis results. The Ohmic resistance (R_{ohm}), interface resistance (R_{cei}) and charge-transfer resistance ($R_{C, CT}$) of pristine $Na_{0.67}Ni_{0.33}Mn_{0.67}O_2$, exposed $Na_{0.67}Ni_{0.33}Mn_{0.67}O_2$ and immersed $Na_{0.67}Ni_{0.33}Mn_{0.67}O_2$ electrodes at the 1st and 100th cycles.

	Pristine sample		Exposed sample		Immersed sample	
	1st cycle	100th cycle	1st cycle	100th cycle	1st cycle	100th cycle
R_{ohm} (Ω)	14	13	13	18	13	19
$R_{C, CT}$ (Ω)	6	26	7	83	6	61
R_{SEI} (Ω)	16	82	17	127	16	99

Supplementary Table 4. Compositions of the hydrated samples. The composition of hydrated phases obtained from the TGA analysis (Supplementary Figure 18-19 and Supplementary Note 7). The processes marked with a^* and b^* correspond to the weight losses of the water extraction from the sodium layers and $NaHCO_3$ decomposition processes, respectively, while c^* stands for the weight losses of the decomposition of Na_2CO_3. It should be point out that it is Na^+/H^+ exchange, rather than O_2 oxidation dominates the charge-compensation mechanisms of Na^+ loss on the basis of the XAS results.

Exposed atmosphere	Pristine samples	Hydrated or not	Weight loss (%)		Corresponding processes	Compositions of the hydrated samples		ICP-AES results
			Total (t^*)	Separate		Based on Na^+-H^+ exchange	Based on O_2 oxidation	$n_{Na} : n_{Ni/Zn/Cu/Al} : n_{Mn}$
RH 18% for 3 days	$Na_{0.67}Ni_{0.33}Mn_{0.67}O_2$	×	0.72	0 (30-130 °C)	---	$Na_{0.646}H_{0.024}Ni_{0.33}Mn_{0.67}O_2$	$Na_{0.645}Ni_{0.33}Mn_{0.67}O_2$	0.651 : 0.335 : 0.665
				0.72 (130-900 °C)	c^*			
RH 93% + CO_2 for 3 days	$Na_{0.67}Ni_{0.33}Mn_{0.67}O_2$	×	5.46	2.71 (30-130 °C)	b^*	$Na_{0.573}H_{0.097}Ni_{0.33}Mn_{0.67}O_2$	$Na_{0.570}Ni_{0.33}Mn_{0.67}O_2$	0.581: 0.327 : 0.673
				2.75 (130-900 °C)	c^*			
RH 93% + CO_2 for 3 days	$Na_{0.67}Zn_{0.2}Mn_{0.8}O_2$	√	24.70	13.61 (30-130 °C)	$a^* + b^*$	$[Na_{0.174}H_{0.496}\cdot(H_2O)_{0.193}]Zn_{0.2}Mn_{0.8}O_2$	$[Na_{0.157}\cdot(H_2O)_{0.164}]Zn_{0.2}Mn_{0.8}O_2$	0.183 : 0.193 : 0.807
				11.09 (130-900 °C)	c^*			
RH 93% + CO_2 for 3 days	$Na_{0.67}Zn_{0.1}Mn_{0.9}O_2$	√	23.70	12.72 (30-130 °C)	$a^* + b^*$	$[Na_{0.189}H_{0.481}\cdot(H_2O)_{0.129}]Zn_{0.1}Mn_{0.9}O_2$	$[Na_{0.174}\cdot(H_2O)_{0.102}]Zn_{0.1}Mn_{0.9}O_2$	0.191 : 0.104 : 0.896
				10.98 (130-900 °C)	c^*			
RH 93% + CO_2 for 3 days	$Na_{0.67}Cu_{0.1}Mn_{0.9}O_2$	√	24.54	13.81 (30-130 °C)	$a^* + b^*$	$[Na_{0.196}H_{0.474}\cdot(H_2O)_{0.234}]Cu_{0.1}Mn_{0.9}O_2$	$[Na_{0.181}\cdot(H_2O)_{0.207}]Cu_{0.1}Mn_{0.9}O_2$	0.208 : 0.107 : 0.893
				10.73 (130-900 °C)	c^*			
RH 93% + CO_2 for 3 days	$Na_{0.67}Al_{0.1}Mn_{0.9}O_2$	√	25.04	14.89 (30-130 °C)	$a^* + b^*$	$[Na_{0.235}H_{0.435}\cdot(H_2O)_{0.350}]Al_{0.1}Mn_{0.9}O_2$	$[Na_{0.220}\cdot(H_2O)_{0.325}]Al_{0.1}Mn_{0.9}O_2$	0.248 : 0.108 : 0.892
				10.15 (130-900 °C)	c^*			
RH 93% + CO_2 for 3 days	$Na_{0.67}MnO_2$	√	23.86	15.10 (30-130 °C)	$a^* + b^*$	$[Na_{0.289}H_{0.381}\cdot(H_2O)_{0.453}]MnO_2$	$[Na_{0.277}\cdot(H_2O)_{0.451}]MnO_2$	0.285 : -- : 1
				8.76 (130-900 °C)	c^*			

Supplementary Table 5. ICP-AES results for prepared oxides.

Oxides	Tm ions	Atomic Ratios
$Na_{0.67}MnO_2$	$n_{Na} : n_{Mn}$	0.675 : 1.000
$Na_{0.67}Al_{0.1}Mn_{0.9}O_2$	$n_{Na} : n_{Al} : n_{Mn}$	0.692 : 0.108 : 0.892
$Na_{0.67}Zn_{0.1}Mn_{0.9}O_2$	$n_{Na} : n_{Zn} : n_{Mn}$	0.673: 0.103 : 0.970
$Na_{0.67}Zn_{0.2}Mn_{0.8}O_2$	$n_{Na} : n_{Zn} : n_{Mn}$	0.679 : 0.194 : 0.806
$Na_{0.67}Ni_{0.33}Mn_{0.66}O_2$	$n_{Na} : n_{Ni} : n_{Mn}$	0.665 : 0.341 : 0.659

Supplementary Notes

Supplementary Note 1

The characterizations of birnessite phase

Besides XRD and NPD, solid-state magic-angle-spinning nuclear magnetic resonance spectroscopy (MAS NMR) is another powerful technique to detect the hydration phases.[2,4,5] As shown in Supplementary Figure 2a, the pristine, partially hydrated (the mixture of pristine and birnessite phases) and totally hydrated (birnessite) $Na_{0.67}MnO_2$ materials have been prepared and their respective ^{23}Na magic-angle-spinning (MAS) NMR spectra are shown in Supplementary Figure 2b. For the pristine $Na_{0.67}MnO_2$, ^{23}Na resonances appear in the region of ~ 1400 ppm as well as at 0 ppm, which could be assigned to the Na^+ in the sodium layers of $Na_{0.67}MnO_2$ and diamagnetic sodium salts (such as Na_2CO_3) on the surface, respectively.[1,5,6] With partial water insertion, a new signal corresponding to Na^+ in the hydration phase appears at ~ 400 ppm, while the $Na_{0.67}MnO_2$ signals at ~ 1400 ppm become weaker and eventually disappears in the totally hydrated sample. The shifting of the Na signal is caused by the reduction of Fermi interaction in the Na^+ ions, because of the expansion of the layer spacings. Moreover, after dehydrating the hydrated sample at 500 °C, the signal at ~ 400 ppm disappears and the $Na_{0.67}MnO_2$ resonances reemerge (Supplementary Figure 3). This confirms that the ^{23}Na signal at ~ 400 ppm belongs to the Na^+ in the hydrated structure, implying that MAS NMR is a reliable technique capable of identifying hydration phases.

So far, both XRD and ^{23}Na MAS NMR characterize the structural transitions, i.e. increased interlayer spacings, rather than the chemical evolutions. While the Fourier-transform infrared spectroscopy (FTIR) and ^1H MAS NMR can be used to detect the existence of water molecules in the hydrated powder sample. Compared to the pristine $Na_{0.67}MnO_2$, the FTIR spectrum of the hydrated sample exhibits a strong O-H stretching band at 2500-3500 cm^{-1} (Supplementary Figure 4a), confirming the insertion of H_2O. Supplementary Figure 4b shows the ^1H MAS NMR spectra from the pristine and hydrated samples, compared with the characteristic signals from NaOH, $NaHCO_3$ and empty rotor. Besides the background signals (from rotor) at 1.24 ppm and 6.87 ppm, the only detectable ^1H signal at 13.4 ppm should be assigned to $NaHCO_3$, indicating

the protons in Hydrated/protonated layered sodium Mn-rich oxides could not be directly observed in ^{1}H NMR spectra. As indicated in Supplementary Figure 2b, the Na^{+} resonances at ~ 400 and ~ 1400 ppm are very broad, due to the effects of unpaired d-orbital electrons of the transition metal Mn. H^{+} has roughly 4-time stronger effects than Na^{+}, provided that their distances to the paramagnetic center are the same. Thus, we would expect that the linewidth for those H^{+} signals are 4-time broader than that of the Na^{+} signals, resulting in a very short spin-spin relaxation, so that most of their signals are decayed during the spin-echo period making their observation extremely difficult, if not impossible.

Supplementary Note 2

$^{23}Na\{^1H\}$ REDOR

We further used the rotational-echo double-resonance (REDOR)[7] technique in MAS NMR to detect hydrogen atoms in the layered oxides. In our $^{23}Na\{^1H\}$ REDOR experiments, the ^{23}Na signals are observed using the rotor-synchronized spin echo sequence, during which a train of ^1H 180° pulses are inserted. The observed ^{23}Na signal intensities are then compared with and without the irradiation of the ^1H 180° pulses. If the Na^+ and hydrogen atoms are far apart, there is no dipolar coupling between the two atoms so that the observed ^{23}Na signals have the same intensities with and without the ^1H irradiation. However, if the Na^+ and hydrogen atoms are in close range, there exists a ^{23}Na-^1H dipolar coupling. As a result, the observed ^{23}Na signals upon ^1H irradiation will be dephased, as compared to that without ^1H irradiation, depending on the ^{23}Na-^1H dipolar coupling. As shown in Supplementary Figure 4c, for pristine $Na_{0.67}MnO_2$, the ^{23}Na signals correspond to the Na^+ in sodium layers (1214 ppm) and diamagnetic Na salts (Na_2CO_3, 0 ppm) remain the same with and without ^1H irradiation, implying that there is no hydrogen atom close to the Na^+ in sodium layers. While for hydrated $Na_{0.67}MnO_2$ (Supplementary Figure 4d), the intensities of both sodium signals of hydrated phase (311 ppm) and diamagnetic salt (0 ppm) decrease with ^1H irradiation, and the attenuation becomes more obvious with the increase of spin echo time from 0.56 to 0.96 ms. These REDOR results confirm the insertion of water molecules and the formation of $NaHCO_3$ on the particle's surface, highly consistent with the FTIR results (Supplementary Figure 4a).

Supplementary Note 3

Immersing in water

As shown in Supplementary Figure 7a, new but weak XRD peaks corresponding to the hydration phase (birnessite, marked with ‡) can be observed in $Na_{0.67}MnO_2$ sample after soaking in water for 15 min, indicating a small amount of $Na_{0.67}MnO_2$ is hydrated. After immersed in water for 20 days, the XRD peaks of pristine $Na_{0.67}MnO_2$ samples disappear, instead, three sets of XRD peaks correspond to birnessite (marked with ‡), buserite (marked with †) and MnOOH (marked with ∇) phases can be recognized, suggesting that the chemical and structural evolutions of P2-Na_xTmO_2 in water exhibit a time-dependent property. As shown in Supplementary Figure 7b, the water-immersed $Na_{0.67}MnO_2$ powder undergoes severe pulverization as compared to the pristine $Na_{0.67}MnO_2$ powder (Figure 2d). Recently, $Na_{0.67}Ni_{0.33}Mn_{0.67}O_2$ was reported to be stable in water for over 1 year. Supplementary Figure 7c and Supplementary Figure 7d show the XRD pattern ranging from 10-80° and 15.2-16.6^o ((002) peak) of $Na_{0.67}Ni_{0.33}Mn_{0.67}O_2$ sample after immersed in water for 20 days, respectively. It can be clearly observed that the (002) peak of this immersed $Na_{0.67}Ni_{0.33}Mn_{0.67}O_2$ sample shifts to a lower 2-theta degree (Supplementary Figure 7d). We also found that the pH value of the water after soaking $Na_{0.67}Ni_{0.33}Mn_{0.67}O_2$ increases gradually from 7.73 to 11.47 (Supplementary Figure 8a), suggesting the presence of the loss and dissolution of Na^+ ions when $Na_{0.67}Ni_{0.33}Mn_{0.67}O_2$ is soaked in water. The SEM image of the soaked $Na_{0.67}Ni_{0.33}Mn_{0.67}O_2$ sample in Supplementary Figure 8b shows that the smooth surface of $Na_{0.67}Ni_{0.33}Mn_{0.67}O_2$ (Figure 2g) is deformed, indicative of the erosion of water to the $Na_{0.67}Ni_{0.33}Mn_{0.67}O_2$ sample. In conclusion, the above results suggest that the structural transformation mechanisms of P2-Na_xTmO_2 in water are similar to that in moisture air, and these changes in water can give new insights into the degradation mechanisms in aqueous batteries.

Supplementary Note 4

TOF-SIMS

As shown in Figure 3a, the $NaHCO_3$ signals disappear after the scavenging process. The TOF-SIMS spectra in Supplementary Figure 9 indicate that there are trace amounts of the carbonate species on the surface of the targeted particles. In the bulk, the intensity of C_2HO^- is ~ 165 a.u., only ~ 2.5 ‰ compared to the intensity of OH^- ions (Figure 3b), indicating that there are nearly no carbon atoms in the bulk of the hydrated samples.

Supplementary Note 5

Protonated phase

As shown in Figure 6, the intensity of XRD patterns in stage *b* is much stronger than stage *c*, suggesting the crystallization of the structure at stage *b* is higher than stage *c*. We speculate that after the extraction of H_2O (70-130 °C, stage *a*), the H^+ ions are extracted from the hydrated sample (stage *b*), and the good crystallization in stage *b* is benefited from the proton ions in the dehydrated $Na_{0.67-x-z}H_xMnO_2$ sample. As shown in Supplementary Figure 15, no hydration signals (311 ppm) and the obvious dephasing in the ^{23}Na-^{1}H REDOR-dephased ^{23}Na ss-NMR spectra suggest that there is a high possibility of the existence of proton in the $Na_{0.67-x-z}H_xMnO_2$ sample in stage *b*, which confirms our speculation.

The sample in Supplementary Figure 15 was annealed as follow: the hydrated powder sample was heated from room temperature (~ 30 °C) to 150 °C by 3 °C per minutes. After annealing the sample at 150 °C for 3 minutes, the dehydrated sample was stored at 120 °C to keep it from re-hydration.

Supplementary Note 6

Verifying the healing effect of high temperature annealing

To verify whether the hydrated materials can be truly fully healed by high temperature. The totally hydrated $Na_{0.67}MnO_2$ samples were annealed at 500 °C and 700 °C for 3 h, separately, and the comparison of their XRD patterns with that of pristine $Na_{0.67}MnO_2$ are presented in Supplementary Figure 17. As shown in Supplementary Figure 17a, it can be observed that all three samples show high crystallinity. However, the comparison of (002) peaks in Supplementary Figure 17b shows that 500 °C-annealed sample exhibit the larger layer spacing than pristine samples, while the location of (002) peaks of pristine and 700 °C-annealed samples are nearly the same. The above results indicate that the hydrated materials are fully recovered at the high temperature of 700 °C.

Supplementary Note 7

TGA analysis

To ascertain the compositions of various hydrated phases, the TGA profiles of $Na_{0.67}Ni_{0.33}Mn_{0.67}O_2$ samples exposed at different atmospheres have been studied firstly, as shown in Supplementary Figure 18a. As we confirmed in Supplementary Figure 11, the degradation products of $Na_{0.67}Ni_{0.33}Mn_{0.67}O_2$ samples exposed at RH 18% and RH 93% + CO_2 atmosphere are Na_2CO_3 and $NaHCO_3$, respectively. In Supplementary Figure 18a, no obvious mass loss at the temperature range of 25-130 °C of $Na_{0.67}Ni_{0.33}Mn_{0.67}O_2$ sample exposed in RH 18% for 15 days can be observed, while the mass loss at the same temperature range of $Na_{0.67}Ni_{0.33}Mn_{0.67}O_2$ sample exposed in RH 93% + CO_2 for 15 days is 2.71 %. In addition, the mass loss at 25-130 °C is similar to that at 130-900 °C, coincides well with the ratio of mass losses at the different stages of $NaHCO_3$ decomposition, as will be shown in Supplementary Note 8.

Combing the above results and the fact that there is no H_2O intercalated into the Na^+ layers of $Na_{0.67}Ni_{0.33}Mn_{0.67}O_2$ samples, we can conclude that: 1) the content of absorbed H_2O molecules at the particle surface is almost negligible, 2) in the exposed samples, $NaHCO_3$ decomposes at the temperature range of 25-130 °C and Na_2CO_3 decomposes at 130-900 °C. Furthermore, according to our in-situ variable-temperature XRD results, the protons are also extracted at 130-900 °C. Therefore, as shown in Supplementary Figure 19, the mass loss of hydrated phases at 25-130 °C and 130-900 °C can be identified to the water deintercalation and $NaHCO_3$ decomposition, and deprotonation plus Na_2CO_3 decomposition, respectively.

Supplementary Note 8

Confirming the compositions of hydrated phases

(1) Na^+-H^+ exchange

30-130 °C

$$2x \cdot NaHCO_3 = x \cdot Na_2CO_3 + \underline{x \cdot H_2O + x \cdot CO_2} \quad (1)$$

$$\frac{m1}{106 \cdot x} \qquad \frac{b^*}{62 \cdot x}$$

$$[Na_{0.67-x}H_x(H_2O)_y]TmO_2 = y \cdot H_2O + [Na_{0.67-x}H_x]TmO_2 \quad (2)$$

$$\frac{a^*}{18 \cdot y} \qquad \frac{m2}{M2}$$

130-900 °C:

$$2[Na_{0.67-x}H_x]TmO_2 + x \cdot Na_2CO_3 = \underline{x \cdot CO_2 + x \cdot H_2O} + 2Na_{0.67}TmO_2 \quad (3)$$

$$\frac{m2}{2 \cdot M2} \qquad \frac{m1}{106 \cdot x} \qquad \frac{c^*}{62 \cdot x} \qquad \frac{100 - a^* - b^* - c^*}{2 \cdot M}$$

$$\frac{m1}{106 \cdot x} = \frac{b^*}{62 \cdot x} = \frac{c^*}{62 \cdot x} = \frac{a^*}{36 \cdot y} = \frac{100 - a^* - b^* - c^*}{2 \cdot M} = \frac{m2}{2 \cdot M2} \quad (4)$$

(2) O_2 oxidation

30-130 °C

$$2x \cdot NaHCO_3 = x \cdot Na_2CO_3 + \underline{x \cdot H_2O + x \cdot CO_2} \quad (5)$$

$$\frac{m1}{106 \cdot x} \qquad \frac{b^*}{62 \cdot x}$$

$$[Na_{0.67-x}(H_2O)_y]TmO_2 = y \cdot H_2O + [Na_{0.67-x}H_x]TmO_2 \quad (6)$$

$$\frac{a^*}{18 \cdot y} \qquad \frac{m2}{M2}$$

130-900 °C:

$$4[Na_{0.67-x}]TmO_2 + 2x \cdot Na_2CO_3 = \underline{2x \cdot CO_2 + x \cdot O_2} + 4Na_{0.67}TmO_2 \quad (7)$$

$$\frac{m2}{4 \cdot M2} \qquad \frac{m1}{212 \cdot x} \qquad \frac{c^*}{120 \cdot x} \qquad \frac{100 - a^* - b^* - c^*}{4 \cdot M}$$

$$\frac{m1}{106 \cdot x} = \frac{b^*}{62 \cdot x} = \frac{c^*}{60 \cdot x} = \frac{a^*}{36 \cdot y} = \frac{100 - a^* - b^* - c^*}{2 \cdot M} = \frac{m2}{2 \cdot M2} \quad (8)$$

M: $Na_{0.67}Ni_{0.33}Mn_{0.67}O_2$ 103.63 g mol^{-1}; $Na_{0.67}Zn_{0.2}Mn_{0.8}O_2$ 104.41 g mol^{-1}; $Na_{0.67}Zn_{0.1}Mn_{0.9}O_2$ 103.41 g mol^{-1}; $Na_{0.67}Cu_{0.1}Mn_{0.9}O_2$ 103.26 g mol^{-1}; $Na_{0.67}Al_{0.1}Mn_{0.9}O_2$ 99.61 g mol^{-1}; $Na_{0.67}MnO_2$ 102.41 g mol^{-1}.

According to the information provided by the FTIR spectra (Supplementary Figure 11) and TGA results (Supplementary Figure 18-19), the content of extracted Na^+ and inserted H_2O in the hydrated phases can be quantified by the above calculating formulas. Moreover, although no direct evidences for O_2 oxidation have been observed, the compositions of the hydrated phases are calculated based on two different compensation mechanisms, e.g. Na^+/H^+ exchange and O_2 oxidation, for the convenience of further studies, as shown in Supplementary Table 4.

Supplementary References

1 Zuo, W. *et al.* Structure-performance relationship of Zn^{2+} substitution in P2–$Na_{0.66}Ni_{0.33}Mn_{0.67}O_2$ with different Ni/Mn ratios for high-energy sodium-ion batteries. *ACS Appl. Energy Mater.* **2**, 4914-4924 (2019).

2 Zuo, W. *et al.* Highly-stable P2–$Na_{0.67}MnO_2$ electrode enabled by lattice tailoring and surface engineering. *Energy Storage Mater.* **26**, 503-512 (2020).

3 Lu, Z. & Dahn, J. R. Intercalation of water in P2, T2 and O2 Structure $A_z[CoxNi_{1/3-x}Mn_{2/3}]O_2$. *Chem. Mater.* **13**, 1252-1257 (2001).

4 Han, M. H. *et al.* Moisture exposed layered oxide electrodes as Na-ion battery cathodes. *J. Mater. Chem. A* **4**, 18963-18975 (2016).

5 Liu, X. *et al.* P2-$Na_{0.67}Al_xMn_{1-x}O_2$: cost-effective, stable and high-rate sodium electrodes by suppressing phase transitions and enhancing Na^+ mobility. *Angew. Chem., Int. Ed.* **58**, 18086-18095 (2019).

6 Clément, R. J. *et al.* Structurally stable Mg-doped P2-$Na_{2/3}Mn_{1-y}Mg_yO_2$ sodium-ion battery cathodes with high rate performance: insights from electrochemical, NMR and diffraction studies. *Energy Environ. Sci.* **9**, 3240-3251 (2016).

7 Gullion, T. & Schaefer, J. Detection of weak heteronuclear dipolar coupling by rotational-echo double-resonance nuclear magnetic resonance. *Advances in Magnetic and Optical Resonance* **13**, 57-83 (1989).
